# Quantifying the regulatory effect size of *cis*-acting genetic variation using allelic fold change

**DOI:** 10.1101/gr.216747.116

**Published:** 2017-11

**Authors:** Pejman Mohammadi, Stephane E. Castel, Andrew A. Brown, Tuuli Lappalainen

**Affiliations:** 1 New York Genome Center, New York, New York 10013, USA;; 2 Department of Systems Biology, Columbia University, New York, New York 10032, USA;; 3 Department of Genetic Medicine and Development, University of Geneva Medical School, Geneva, 1211, Switzerland;; 4 Institute for Genetics and Genomics in Geneva (iGE3), University of Geneva, Geneva, 1211, Switzerland;; 5 Swiss Institute of Bioinformatics, Geneva, 1211, Switzerland

## Abstract

Mapping *cis*-acting expression quantitative trait loci (*cis*-eQTL) has become a popular approach for characterizing proximal genetic regulatory variants. In this paper, we describe and characterize log allelic fold change (aFC), the magnitude of expression change associated with a given genetic variant, as a biologically interpretable unit for quantifying the effect size of *cis*-eQTLs and a mathematically convenient approach for systematic modeling of *cis*-regulation. This measure is mathematically independent from expression level and allele frequency, additive, applicable to multiallelic variants, and generalizable to multiple independent variants. We provide efficient tools and guidelines for estimating aFC from both eQTL and allelic expression data sets and apply it to Genotype Tissue Expression (GTEx) data. We show that aFC estimates independently derived from eQTL and allelic expression data are highly consistent, and identify technical and biological correlates of eQTL effect size. We generalize aFC to analyze genes with two eQTLs in GTEx and show that in nearly all cases the two eQTLs act independently in regulating gene expression. In summary, aFC is a solid measure of *cis*-regulatory effect size that allows quantitative interpretation of cellular regulatory events from population data, and it is a valuable approach for investigating novel aspects of eQTL data sets.

Noncoding genetic variation affecting gene regulation and other cellular phenotypes has a key role in phenotypic variation and disease susceptibility (Albert and Kruglyak 2015). One of the most commonly used methods to characterize genetic variants that affect gene expression is expression quantitative trait loci (eQTL) mapping (Schadt et al. 2003; Lappalainen et al. 2013; The GTEx Consortium 2015), which identifies genetic loci where genotypes of genetic variants are significantly associated to gene expression in a population sample. Genes and variants with significant associations are often called eGenes and eVariants, respectively, and the eVariant with the best *P*-value in a given locus usually used as the proxy for the causal variant. The association between genotype and gene expression is typically tested by regressing gene expression on the number of alternative alleles using a linear model, and the significance of the regression slope is used to measure significance of the eQTL (Shabalin 2012; Ongen et al. 2016). eQTLs can occur either in *trans* through altering diffusible factors that affect gene expression distally or in *cis* through allelic, physical interactions on the same chromosome typically <1 Mb away from the eGene, which are the focus of this study. The allelic effect of *cis*-regulation leads to unequal expression of the two haplotypes (allelic imbalance) in individuals that are heterozygous for a *cis*-acting eVariant (Fig. 1A).

**Figure 1.**
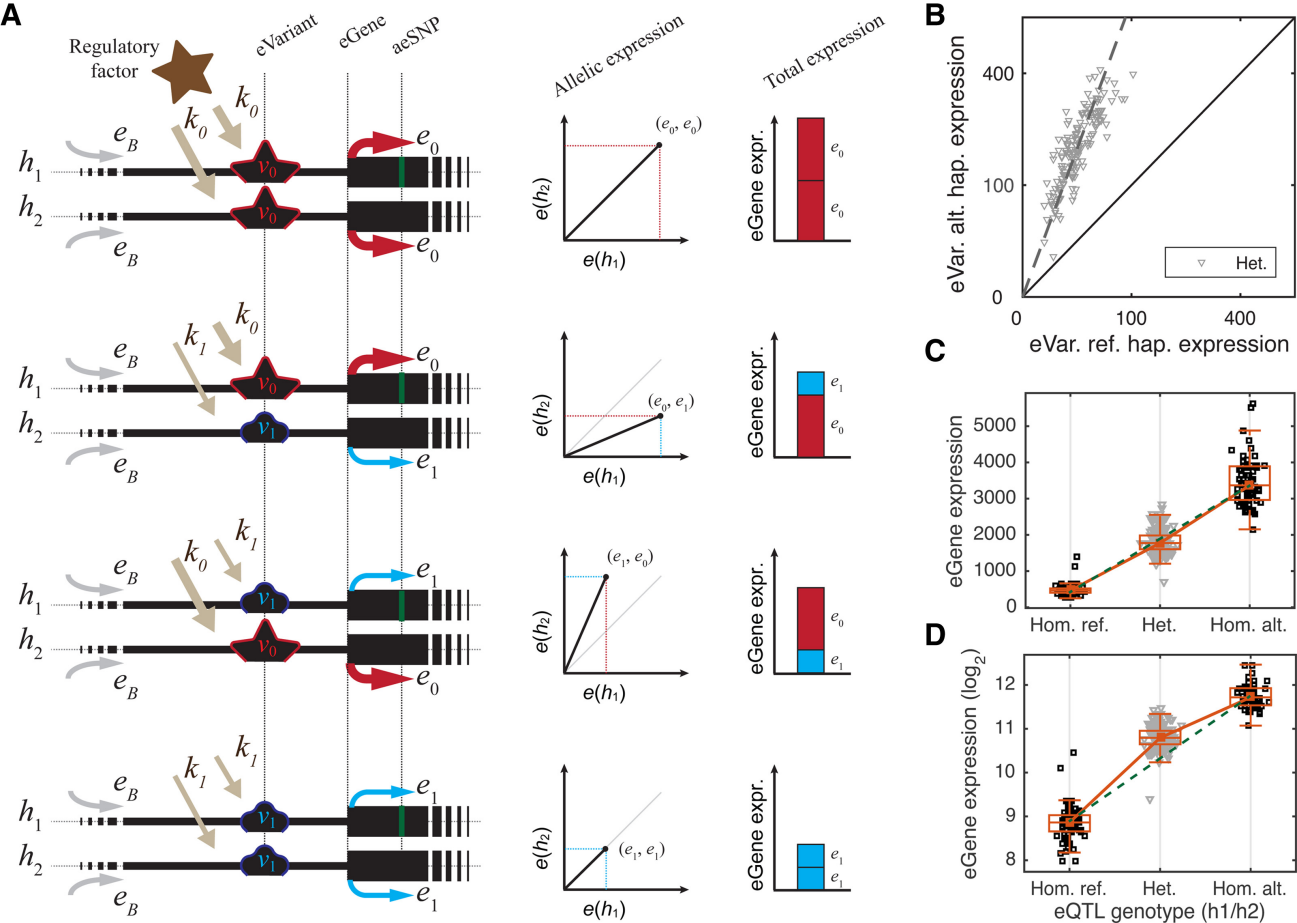
(*A*) Schematic representation of *cis*-regulatory eQTL model in Equations 1 and 2. (*B*) Example of allelic expression associated with each of the alleles of a *cis*-eQTL (eVariant Chr 5: 96252589 T/C; eGene *ERAP2*) in GTEx adipose subcutaneous. Each dot corresponds to allelic imbalance in one individual heterozygous for the eVariant, measured using reads that overlap heterozygous SNPs (aeSNP) in the eGene. Phasing between the aeSNP and the eQTL SNP is utilized to associate the measured allelic expression with each of the eQTL alleles. (*C*,*D*) eGene expression for the same example eQTL. The green dashed line connects the median expression of the two homozygous classes. Expression is linear with number of alternative alleles (*C*), but the linearity is lost after log transformation (*D*).

The effect size of an eQTL describes the magnitude of the effect that it has on gene expression and is an important statistic for characterizing the nature of regulatory variants. Estimating the relative effect of eQTL alleles on expression levels has applications in computational functional genetics analysis, as well as in analysis of genetic regulatory variants by experimental assays such as genome editing (Arnold et al. 2013; Canver et al. 2015; Vockley et al. 2015; Tewhey et al. 2016; Ulirsch et al. 2016; Wright and Sanjana 2016). However, thus far there has been no consensus definition for eQTL effect size, with each study defining its own measure for quantifying regulatory effect size. The most widely used measure of effect size is the linear regression slope, a readily available statistic from eQTL calling tools (Shabalin 2012; Gutierrez-Arcelus et al. 2013; Lee et al. 2015; Tung et al. 2015). Linear regression has also been utilized on log-transformed (Flutre et al. 2013; Battle et al. 2014, 2015) or *z*-scored expression data (Lappalainen et al. 2013) to derive slope estimates that do not depend on expression levels. Other statistics include the observed difference between genotype classes, such as the mean difference in expression between heterozygous and the more common homozygote class, sometimes with log transformation or scaling by mean (Gutierrez-Arcelus et al. 2015; Josephs et al. 2015). The proportion of expression variance in the population explained by an eQTL is a widely used statistic that is informative of population variance but not of the molecular effect of an eQTL (Grundberg et al. 2012; Wright et al. 2014; Kirsten et al. 2015). A recent method, developed simultaneously and independently from our work, uses the ratio between the slope and intercept of the linear regression in a variance stabilized model (Palowitch et al. 2016). While all these approaches provide estimates that are generally correlated with *cis*-regulatory effect of a given variant and have a specific statistical interpretation in the context of eQTL data, they often lack a straightforward biological interpretation in the greater context of *cis*-regulation that is comparable across different studies, conditions, or data types. Furthermore, many of these easily accessible statistics systematically depend on other variables such as gene expression level, allele frequency, or the amount of technical variation or other noise, which hinders their broad usability across different studies. Finally, a group of statically involved *cis*-eQTL calling methods include regulatory effect size as one of the many parameters for the models that map regulatory variants using both allele-specific expression (ASE) and total gene expression data (Pickrell et al. 2010; Sun 2012; Hu et al. 2015; van de Geijn et al. 2015; Kumasaka et al. 2016), but these methods are distinct from standard, commonly used methods for *cis*-eQTL mapping.

In this study, based upon the mechanistically justified model of *cis* genetic effects on gene expression, we advocate using the log-ratio between the expression of the haplotype carrying the alternative allele to the one carrying the reference allele, the log *allelic fold change* (aFC), as a biologically interpretable and mathematically convenient measure of *cis-*regulatory effect size, applicable to eQTLs discovered by standard eQTL calling methods. This measure is equivalent to the expected log-fold expression ratio of the individuals homozygous for the alternative allele to those homozygous for the reference allele of an eQTL. We provide a thorough description of the derivation and properties of aFC and its generalizations, present practical guidelines and tools for calculating aFC from eQTL as well as allelic expression data, and demonstrate how the extended aFC model can be applied to study more complex regulatory scenarios.

## Results

### Model

#### Additive model of regulation

For a given gene and a given *cis*-regulatory variant, *v*, with two alleles in the population, *v*_0_ and *v*_1_, we define allelic expressions *e*_0_, and *e*_1_ as the amount of transcript produced from the gene when it is located on the same chromosome copy as alleles *v*_0_, and *v*_1_, respectively. We assume that the allelic expression is determined by a shared basal expression of the gene, *e*_*B*_, driven by the cellular regulatory environment, and allele-specific factors *k*_0_, *k*_1_ ≥ 0, which represent distinctive effect of the allele *v*_0_, and *v*_1_ on transcription, respectively (Fig. 1A):

(1) $$\begin{array}{c} e_{0}=k_{0}e_{B},\\ e_{1}=k_{1}e_{B}. \end{array}$$

Under the *cis*-regulatory model, the regulatory effect of an allele does not depend on the genotype on the other chromosome copy, and *e*_*i,j*_, the total expression of the gene in an individual with alleles *v*_*i*_ and *v*_*j*_ on the first and second haplotype is

(2) $$e_{i,j}=(k_{i}+k_{j})e_{B},\;i,j\in \{0,1\}.$$

By using δ_i,j_ = *k*_i_/*k*_j_ in Equation 1, the expression of haplotype carrying the alternative allele *v*_1_ is given as

(3) $$e_{1}=\delta_{1,0}e_{0},$$

relative to *e*_0_, the expression of the haplotype carrying the reference allele. Similarly, the total relative expression of the gene is

(4) $$e_{i,j}=(\delta_{i,0}+\delta_{j,0})e_{0},\;i,j\in \{0,1\}.$$

For a given *cis*-acting eVariant, we define log aFC, $s_{1,0}=\log_{2}\delta_{1,0}$, as the relative *cis*-regulatory strength of the allele *v*_1_ versus the reference allele *v*_0_. This quantity is similar to the widely used log expression fold change of differentially expressed genes, but defined between two alleles of a genetic variant. The aFC of a biallelic eVariant can be directly quantified from allelic gene expression in heterozygous individuals (Fig. 1A,B; Supplemental Fig. S1; Box 1) or from summary statistics of standard eQTL linear regression between genotypes and total expression levels (Fig. 1C; Box 2). A further challenge in eQTL effect size estimation is the heteroscedasticity of noise in expression data, which violates the data normality assumptions of linear regression. Although different RNA measurement platforms such as RNA sequencing, microarrays, and other techniques have distinct technical variation profiles, biological variation in gene expression data is generally considered to be log-normally distributed (Tu et al. 2002; Whitehead and Crawford 2006; Anders and Huber 2010). However, after the commonly used variance stabilization by log transformation, gene expression is no longer a linear function and, as such, cannot be characterized efficiently (Fig. 1D; Methods). Thus, we introduce an efficient approximation method to estimate aFC from log-transformed total gene expression data in linear time (Box 3; Methods). The method generates a set of four candidate aFC estimates: The first three estimates are calculated by using only two out of the three eQTL genotype classes at a time. The fourth estimate is derived using log-linear regression, utilizing the fact that log-transformed eQTL data approach a linear function in weak eQTLs as log aFC goes to zero (*s*_1,0_ → 0; Methods). The candidate aFC that minimizes the residual variance in log-transformed data is reported as the final estimate (Methods).

Box 1. **Calculating aFC from allelic expression data. Allelic expression associated with each of the eQTL alleles can be measured in individuals that are heterozygous for the eQTL and that are heterozygous for at least one variant in the eGene (aeSNP). Since allelic expression is measured at the aeSNPs, haplotype phasing data are utilized to obtain the allelic expression from each of the eQTL alleles (Supplemental Fig. S1).**

Input:

- Allelic expression of the haplotypes carrying the reference (*c*_0,n_), and the alternative allele (*c*_1,n_) of an eQTL in *N* individuals: (*c*_0,n_, *c*_1,n_), where *n* ∈ {1,2,…, *N*}

1. Get median ratio of the allelic counts:
  $$\delta_{1,0}=\underset{n=1\ldots N}{\text{median}}\frac{c_{1,n}}{c_{0,n}}.$$

Output: Report effect size: *s*_1,0_ = log_2_ δ_1,0_

Box 2. **Calculating aFC from gene expression data (for derivations, see Methods).**

Input:

- eGene expression in *N* individuals: *y*_1_ … *y*_N_, where y_n_ ∈ [0, +∞)
- Number of alternative alleles in each individual: *t*_1_ … *t*_N_, where *t*_n_ ∈ {0,1,2}

1. Use simple linear regression to model expression as a function of *t*_n_:
  $$y_{n}=b_{0}+b_{1}t_{n}+\text{noise}.$$
2. Use the slope *b*_1_ and intercept *b*_0_ to calculate
  $$\delta_{1,0}=\frac{2b_{1}}{b_{0}}+1.$$

Output: Report effect size: *s*_1,0_ = log_2_ δ_1,0_

Box 3. **Linear time algorithm for estimating aFC from log-transformed gene expression data (for derivations, see Methods).**

Input:

- eGene expression in *N* individuals in log_2_ scale: *z*_1_ … *z*_N_, where *z*_n_ ∈ [–∞, +∞)
- Number of alternative alleles in each individual: *t*_1_ … *t*_N_, where *t*_n_ ∈ {0,1,2}

1. Calculate *m*_0_, *m*_1_, *m*_2_ as geometric mean of expression for individuals with *t*_*n*_ = 0, 1, and 2, respectively.
2. Calculate the following three candidate estimates:
  $$\begin{array}{c} \delta_{1,0}^{*1}=\frac{m_{2}}{m_{0}}\\ \delta_{1,0}^{*2}={\left(2\frac{m_{1}}{m_{2}}-1\right)}^{-1}\\ \delta_{1,0}^{*3}=2\frac{m_{1}}{m_{0}}-1 \end{array}$$
3. Use simple linear regression to model log_2_ expression as a function of *t*_*n*_:
  $$z_{n}=c_{1}t_{n}+c_{0}+\text{noise}.$$
4. Use the slope *c*_*1*_ times two as the fourth candidate estimate:
  $$\delta_{1,0}^{*4}=2^{2c_{1}}.$$
5. Use each of the four estimates $\delta_{1,0}^{*i},\;k=1\ldots 4$ to calculate
  $$r_{n}(i)=z_{n}-\log_{2}\left[(2-t_{n})+t_{n}\delta_{1,0}^{*i}\right],$$
  where $(2-t_{n})+t_{n}\delta_{1,0}^{*i}$ is predicted gene expression in *n*th individual using the *i*th estimate.
6. Pick the estimate that provides the lowest variance in the residuals:
  $$\delta_{1,0}=\delta_{1,0}^{*I},\;I=\underset{i\in 1\ldots 4}{\text{argmin}}\text{V[}r(i)].$$

Output: Report effect size: *s*_1,0_ = log_2_ δ_1,0_

#### Generalization to multiple eVariants with multiple alleles

Beside clear biological interpretation, log aFC has several convenient mathematical properties that facilitate downstream analysis of the values (Box 4, Supplemental Methods) and allow generalization to analysis of multiallelic genetic variants, as well as to joint analysis of multiple independent eQTLs for the same eGene. Here we consider the case of *N* eVariants, *v*1, …, *v*n, …*v*N acting on the same eGene independently with *m*_1_, … *m*_n_, … *m*_N_ alleles, respectively. Let *i*_1_…*i*_n_…*i*_N_ denote a haplotype carrying the *i*_n_-th allele of the *v*n; the relative expression on this haplotype is

(5) $$e_{i_{1}\ldots \;i_{n}\ldots i_{N}}=e_{0}\overset{N}{\underset{n=1}{\prod }}\delta_{i_{n},0}^{vn},$$

where $\delta_{i_{n},0}^{vn}$ denotes the aFC associated with allele *i*_n_, at the *n*th eVariant *v*n versus its reference allele 0, and *e*_0_ is the reference expression associated with the case *e*_0… 0…0_, where the haplotype carries reference alleles for all eVariants. Thus, the log allelic fold difference between two haplotypes *i*_1_… *i*_*n*_…*i*_*N*_ and *j*_1_… *j*_*n*_…*j*_*N*_ is

(6) $${\text{s}}_{i_{1}\ldots \;i_{n}\ldots i_{N},\;j_{1}\ldots \;j_{n}\ldots j_{N}}=\overset{N}{\underset{n=1}{\sum }}{\text{s}}_{i_{n},j_{n}}^{vn},$$

where ${\text{s}}_{i_{n},j_{n}}^{vn}$ denotes the log aFC associated with two alleles *i*_n_ and *j*_n_, at the *n*th eVariant. The total expression of the eGene given the genotype is

(7) $$e_{i_{1}\ldots \;i_{n}\ldots i_{N},\;j_{1}\ldots \;j_{n}\ldots j_{N}}=e_{0}\left(\overset{N}{\underset{n=1}{\prod }}\delta_{i_{n},0}^{vn}+\overset{N}{\underset{n=1}{\prod }}\delta_{\;j_{n},0}^{vn}\right).$$

Box 4. **Mathematical properties of log aFC as a relative measure of *cis*-regulatory effect size (for proofs, see Supplemental Methods).**

1. Zero log aFC indicates the absence of regulatory difference: *s*_*i*,*i*_ = 0.
2. Choice of reference allele only affects the sign of log aFC: *s*_*i*,*j*_ = −*s*_*j*,*i*_.
3. Log aFC is additive:
  $$s_{i,k}=s_{i,j}+s_{\;j,k}.$$
4. Log aFC associated with joint effect of independent regulatory variants, *v*1…*v*N is sum of their individual aFCs:
  $${\text{s}}_{i_{1}\ldots \;i_{n}\ldots i_{N},\;j_{1}\ldots \;j_{n}\ldots j_{N}}=\overset{N}{\underset{n=1}{\sum }}{\text{s}}_{i_{n},j_{n}}^{vn},$$
  where *i*_1_… *i*_*n*_…*i*_*N*_ and *j*_1_… *j*_*n*_…*j*_*N*_ are the set of present alleles on each of the haplotypes.
5. Absolute value of log aFC, *d*_*i*,*j*_ = |*s*_*i*,*j*_|, is a pseudometric:
  - *d*_*i*,*j*_ ≥ 0,
  - *d*_*i*,*i*_ = 0,
  - *d*_*i*,*j*_ = *d*_*j*,*i*_,
  - *d*_*i*,*k*_ ≤ *d*_*i*,*j*_ + *d*_*j*,*k*_.

Following the *cis*-regulatory model, this inherently takes specific configuration of the alleles on each of the two haplotypes into account. The last two equations can be used to simultaneously estimate effect sizes of *N* eVariants from allelic expression or transcription profiles of genotyped individuals, respectively.

### Calculating aFC

**Figure 2.**
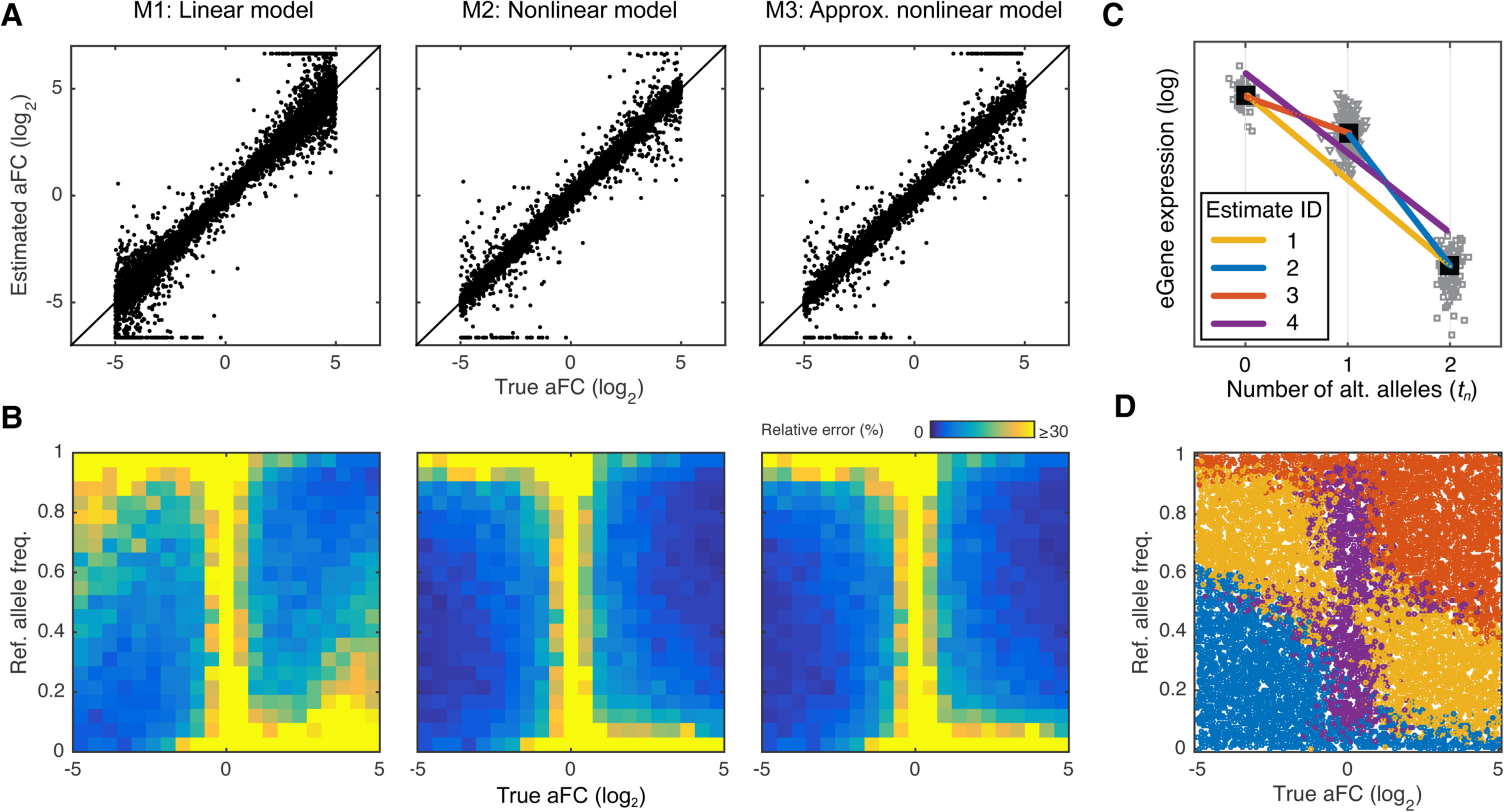
Comparison of the aFC estimation methods using simulated data. We simulated 10,000 eQTLs with noise (40% coefficient of variation), and uniformly selected log_2_ aFC (range: [−5,5]), and reference allele frequency (range: [0,1]). (*A*) True aFC used in simulation versus identified values using linear model (M1), nonlinear model (M2), and the nonlinear model approximation (M3). At this level of noise, M2 performed the best, with M1 and M3 having RMSDs of 164% and 110% of M2. (*B*) Quality of the effect size estimates as a function of allele frequency and the true effect size, evaluated by average error relative to the true log_2_ aFC. All three estimates, and particularly M1, deteriorate when the lower expressed allele is the minor allele. (*C*,*D*) Schematic representation of the nonlinear model approximation method (Box 3) based on four different candidate estimates (*C*), and the selected estimate with minimum residual variance for each simulated eQTL as a function of reference allele frequency and the true aFC (*D*).

We used simulation to evaluate how our three alternative methods for calculating aFC perform under a realistic expression noise level: M1, linear method that uses linear regression coefficients from eQTL data as benchmark for speed (Box 2); M2, nonlinear method that directly solves the regression problem in Equation 17 using a standard nonlinear least square optimization tool (Methods) as a benchmark for accuracy; and M3, nonlinear approximation that solves the nonlinear regression problem from Equation 17 in linear time (Box 3, Fig. 2C). In this simulation, we used simulated data of 10,000 eQTLs with varying allele frequencies and effect sizes (Equations 3, 4), with noise added to the expression levels at 40% coefficient of variation within genotype groups (log_10_ ε_n_ ∼ *norm*[0, σ = 0.17]; Equation 17) similar to what is observed in real data from GTEx (Supplemental Fig. S2). We found that at this level of noise all three methods provide highly accurate and similar estimates (Fig. 2). All estimates, especially the linear method (M1), deteriorate in eQTLs in which the lower expressed allele has also a low frequency (Fig. 2B). This problem is inherent to *cis*-eQTL data and is expected to occur regardless of the expression measurement platform. Overall, all three methods achieved comparable performances. Specifically, the aFC estimates from the nonlinear model (M2) provided the lowest root mean squared deviation (RMSD) from the true values. The linear model was 84 times faster than the nonlinear model but provided 64% higher RMSD. Finally, the nonlinear approximation (M3) presented a trade-off between the speed and accuracy, providing only 10% higher RMSD than the nonlinear model at only 1.8 times the runtime of the linear model.

**Figure 3.**
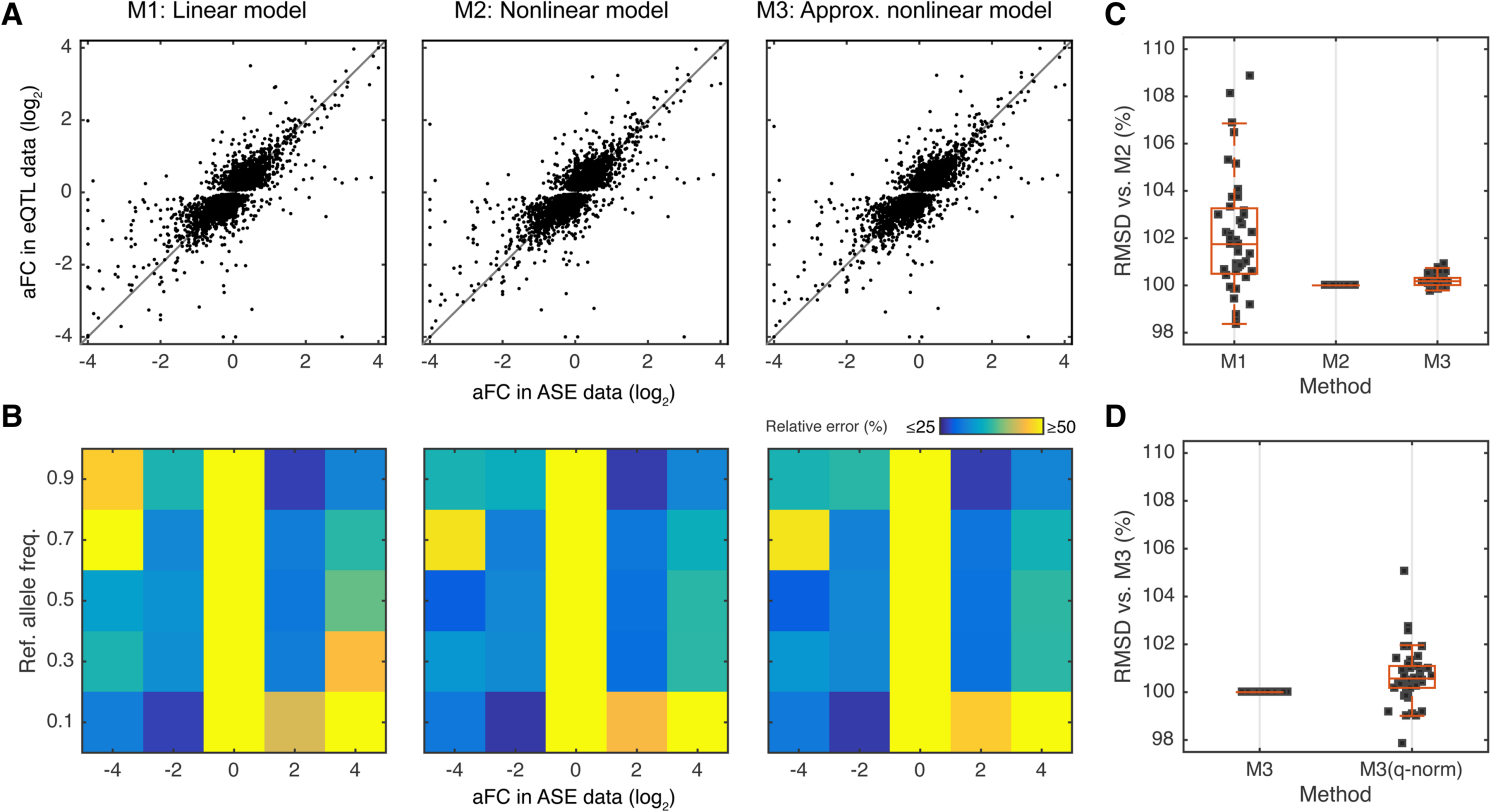
Comparison of the methods for estimating aFC using GTEx data. (*A*) aFC as estimated from ASE data versus estimates from eQTL data using linear model (M1), nonlinear model (M2), and the nonlinear model (M3) approximation for all top eQTLs in adipose subcutaneous. All three estimates are ∼75% correlated with estimates from ASE data. (*B*) Quality of the eQTL estimates as a function of allele frequency and the aFC estimate from allelic expression data, evaluated by average relative error between aFC from ASE data and from eQTL estimates. (*C*) Concordance between the estimates from allelic expression and eQTL data as evaluated by RMSD between the most accurate method, M2, and the other two methods. Each dot represents one tissue in GTEx. (*D*) Concordance between the estimates from ASE and eQTL data as evaluated by RMSD, comparing M3 to M3 applied after quantile normalization within each genotype group. Each dot represents one tissue in GTEx.

Next, we applied the three methods for effect size estimation to the *cis*-eQTLs discovered in the Genotype Tissue Expression (GTEx) (The GTEx Consortium 2013, 2015) v6p data set, with eQTL data from 44 tissues (70 to 361 individuals per tissue) (The GTEx Consortium 2017), calculating aFC for all the reported eQTLs in each tissue using the eVariant with the best *P*-value for each eGene. aFCs were estimated from both ASE (Box 1) and eQTL data (Boxes 2–3), independently. For ASE data, we used haplotypic expression at eGenes calculated by summing allelic expression from all phased heterozygous SNPs within the gene (Supplemental Fig. S1). aFC was reported for an average of 57% of eGenes per tissue, requiring haplotypic coverage of at least 10 reads in at least five individuals (The GTEx Consortium 2017). For eQTL-based aFC estimates, we log transformed normalized read counts and corrected for significant linear effects by confounding factors identified using PEER (Stegle et al. 2012) and the top three principal components of the genotype matrix (see Methods, Equations 23, 24). The log aFCs for the eQTLs were calculated using the three models as in the simulation study and constrained to ±log 100. All three eQTL methods provided highly similar aFC estimates with high concordance to ASE-based estimates (Fig. 3A,C). The effect sizes were more discordant between ASE- and eQTL-based estimates when the rare allele was the lower expressed allele, as predicted by the simulation study (Fig. 3B). The nonlinear model provided the best estimates as evaluated by RMSD from ASE-based estimates, and was closely trailed by the nonlinear approximation method (Fig. 3C). Thus, for the rest of the analyses, we used only the nonlinear approximate method as it provided both high accuracy and speed.

Accounting for confounding variation by methods such as PEER is commonly used to improve the statistical power in eQTL calling. Next, we evaluated the effect of this correction on aFC estimates from eQTL data (Supplemental Fig. S3). We found that it has a minimal impact on the aFC estimates (Pearson R = 0.96). However, correcting for confounding sources of variation leads to narrower confidence intervals for the aFC estimates, which is consistent with the increased power in eQTL calling. Finally, we tested the effect of quantile normalization that enforces log-normality of expression data within each genotype. While this is commonly used to avoid outlier effects, we did not observe improvement of the effect size estimates (Fig. 3D).

### Comparison of aFC to slope of linear regression

Linear regression slope is the most common measure used for estimating *cis*-eQTL effect size. aFC is closely related to this familiar statistic. From an analytical point of view, the aFC estimation method presented in Box 2 (M1) is a normalization technique to appropriately account for gene expression level. Furthermore, the nonlinear eQTL model provided for estimating aFC from log-transformed gene expression data (M2; Equation 17) is well approximated by log-linear regression for weak eQTLs (for proof, see Supplemental Information). In this case, the regression slope is approximately half of the log aFC; a property we used in the nonlinear approximation method provided in Box 3 (M3) to derive one of the four candidate aFC estimates (Fig. 2C,D).

**Figure 4.**
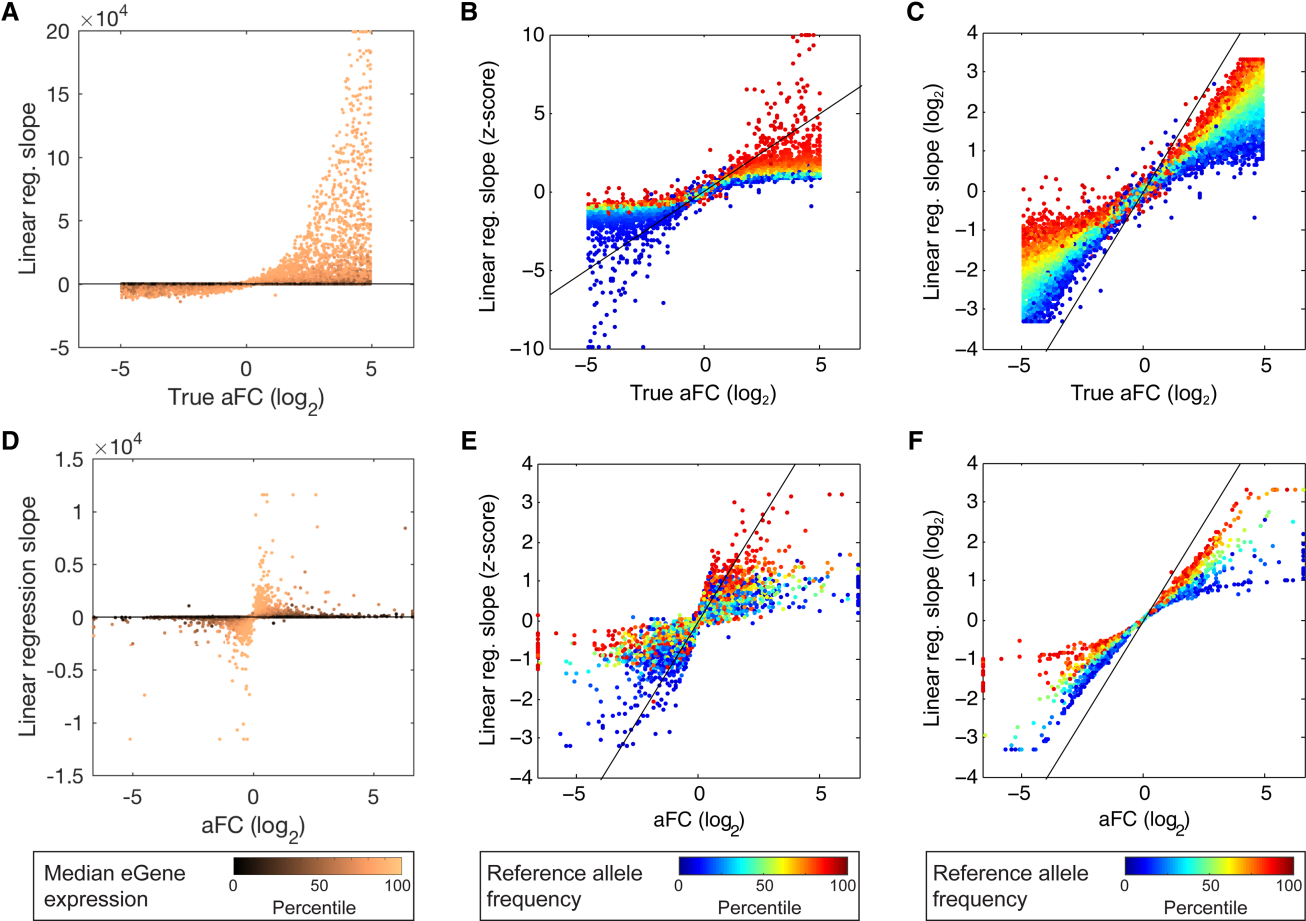
aFC compared with linear regression slope. (*A*–*C*) Slope of linear regression from 10,000 simulated eQTLs generated similarly to data shown in Figure 2. The true aFC value is compared with regression slopes from raw (*A*), *z*-scored (*B*), and log_2_ transformed (*C*) data. The color code represents median eGene expression (*A*) and reference allele frequency (*B*,*C*) with alternative color-coding for the same plots in Supplemental Figure S4. (*D*–*F*) Regression slope compared with aFC values estimated using GTEx eQTLs data from adipose subcutaneous.

We used the simulations with realistic expression noise described above to compare the slope of linear regression to aFC. In addition to using linear regression on untransformed expression data, we considered log transformation and *z*-scoring as two common approaches used for eliminating the effect of gene expression level on regression estimates. The results demonstrated that the two transformations largely remove the effect of gene expression level from regression slopes and yield estimates that are highly correlated with aFC estimates (Fig. 4A–C; Supplemental Fig. S4). However, in both cases the transformation introduces systematic biases in the effect size estimates that manifest as distinct deviation patterns from the simulated aFC with respect to allele frequency and eQTL strength. Specifically, in log-transformed data, the slope of linear regression is skewed proportional to frequency of the lower expressed allele, and in *z*-scored data, the slope is inflated as allele frequency deviates from 50% (Fig. 4A–C; Supplemental Fig. S5). We used GTEx data from adipose subcutaneous to see if these biases can be observed in real data using aFC estimates as a baseline. This analysis recapitulated the patterns observed in simulations (Fig. 4D–F). Altogether, these results show that while regression slope is a useful statistic for many purposes, its direct use as eQTL effect size leads to suboptimal results compared with aFC.

### Application to GTEx eQTLs

**Figure 5.**
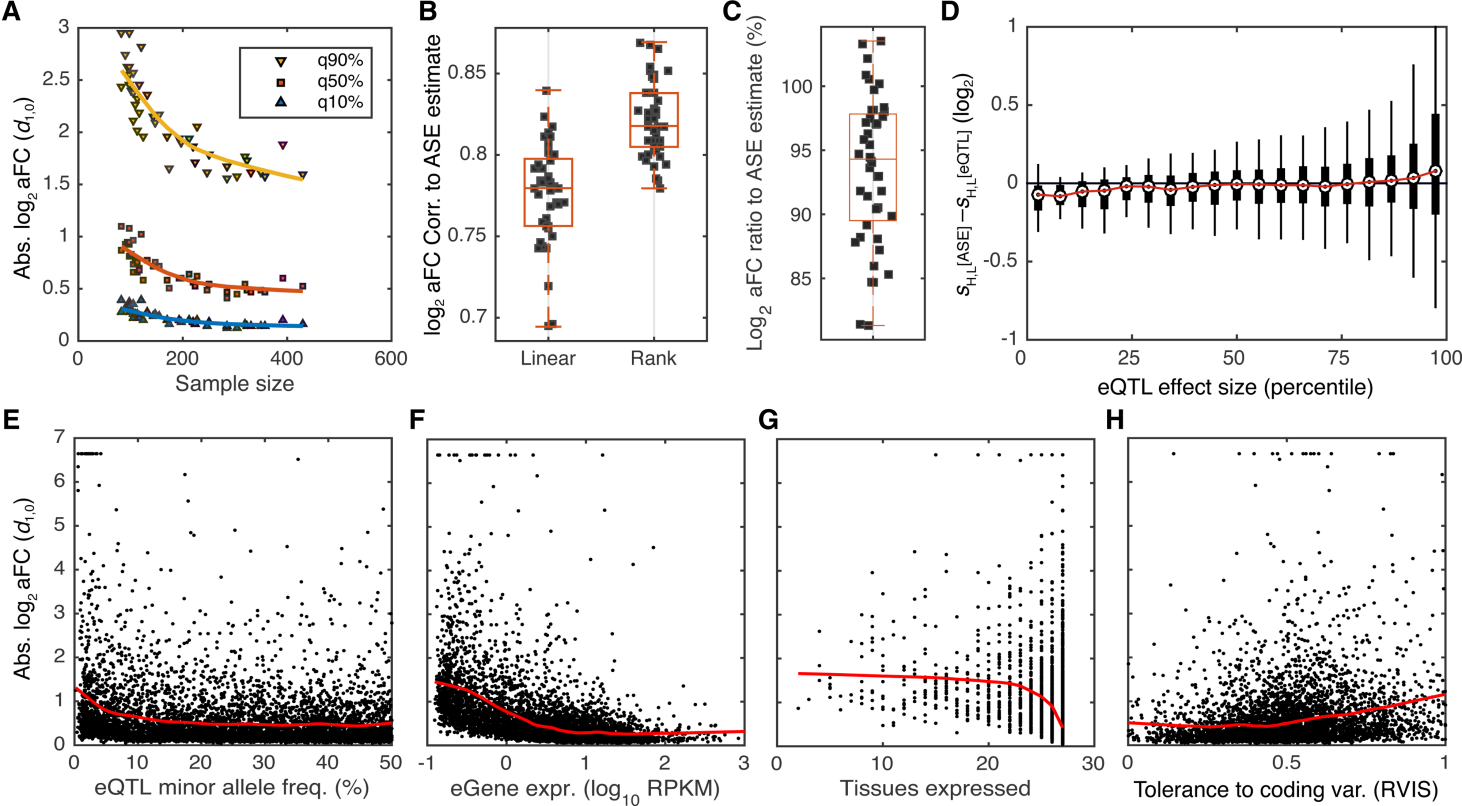
Empirical properties of the aFC distributions in GTEx data. All aFC values are calculated with the nonlinear approximation method (M3). (*A*) Distribution of absolute log_2_ aFC across tissues as a function of sample size. Each point represents a tissue in GTEx data, and 90%, 50%, and 10% quantiles of absolute aFC across a tissue are shown. (*B*,*C*) Correlation of log_2_ aFC estimates (*B*), and the ratio of the estimates (*C*) derived from eQTL and ASE data. Each point corresponds to one GTEx tissue. (*D*) Difference between the aFC estimates from allelic expression (*s*^ASE^) and eQTL (*s*^eQTL^) as a function of absolute average aFC (|*s*^ASE^_+_ *s*^eQTL^|/2), with H and L referring to higher and lower expressed alleles of each eQTL in adipose subcutaneous, respectively. Estimated effect size form ASE data tend to be smaller in weak eQTLs and larger for stronger eQTLs as compared to those derived using eQTL data. (*E–H*) Distribution of absolute log_2_ aFCs calculated from GTEx adipose subcutaneous as function of minor allele frequency (*E*), gene expression level (*F*), number of tissues where the gene is expressed >0.1 RPKM in 10 or more individuals (*G*), and logistic-transformed RVIS, a measure of each gene's tolerance to variation in the coding region (*H*) (Petrovski et al. 2013). Red line shows fit by robust locally weighted scatterplot smoothing.

Next, we used GTEx data to explore empirical properties and general trends in eQTL effect size data measured by log aFC. We found that the distributions of aFCs for eQTLs detected in different GTEx tissues are highly dependent on the sample size, due to the fact that tissues with lower sample size lack the power to detect weak eQTLs (Fig. 5A). The effect size estimates from eQTL and ASE data are highly similar but overall 6.35% smaller (CI: [4.6, 8.1]; estimated by errors-in-variables linear regression fit) across the tissues when estimated from eQTL data (Fig. 5B,C). However, this pattern is reversed in effect sizes involving weaker eQTLs, which is consistent with potential winner's curse in the eQTL calling stage (Fig. 5D). This highlights the added value of ASE-based estimates alongside eQTL data. We next analyzed the correlation of aFC with other properties of the eVariant or eGene. Low-frequency eVariants tend to have higher effect sizes (Fig. 5E), likely a compound effect of increased selection pressure on stronger eQTLs as well as reduced statistical power in calling weak low-frequency eQTLs with limited data. eGenes with high expression levels, expression in multiple tissues, and high coding region conservation measured by RVIS (Petrovski et al. 2013) have lower effect sizes (Fig. 5F–H), which suggests that genes under strong selective constraints are less likely to tolerate regulatory variants with high effect sizes. Further biological interpretation of effect sizes across eVariants in different annotations, eGenes of different biotypes, and eQTLs that are tissue specific or shared is described by The GTEx Consortium (2017). In these and other downstream analyses of eQTL effect sizes, it is important to correct for correlated factors such as sample size and allele frequency. Even though our simulations demonstrate that aFC is highly robust to key confounders, differences in the power of eQTL mapping will always affect the properties of discovered eQTLs, including effect size distribution.

The aFCs of GTEx eQTLs are provided in the GTEx portal (http://gtexportal.org). Additionally, we implemented the linear model (M1) and the nonlinear approximation model (M3) in a python script (see Software Availability) that takes as input the standard file formats used also by the FastQTL software for eQTL calling. This makes calculation of aFC for other eQTL data sets straightforward and fast.

### Application to genes with two distinct eQTL signals in GTEx

**Figure 6.**
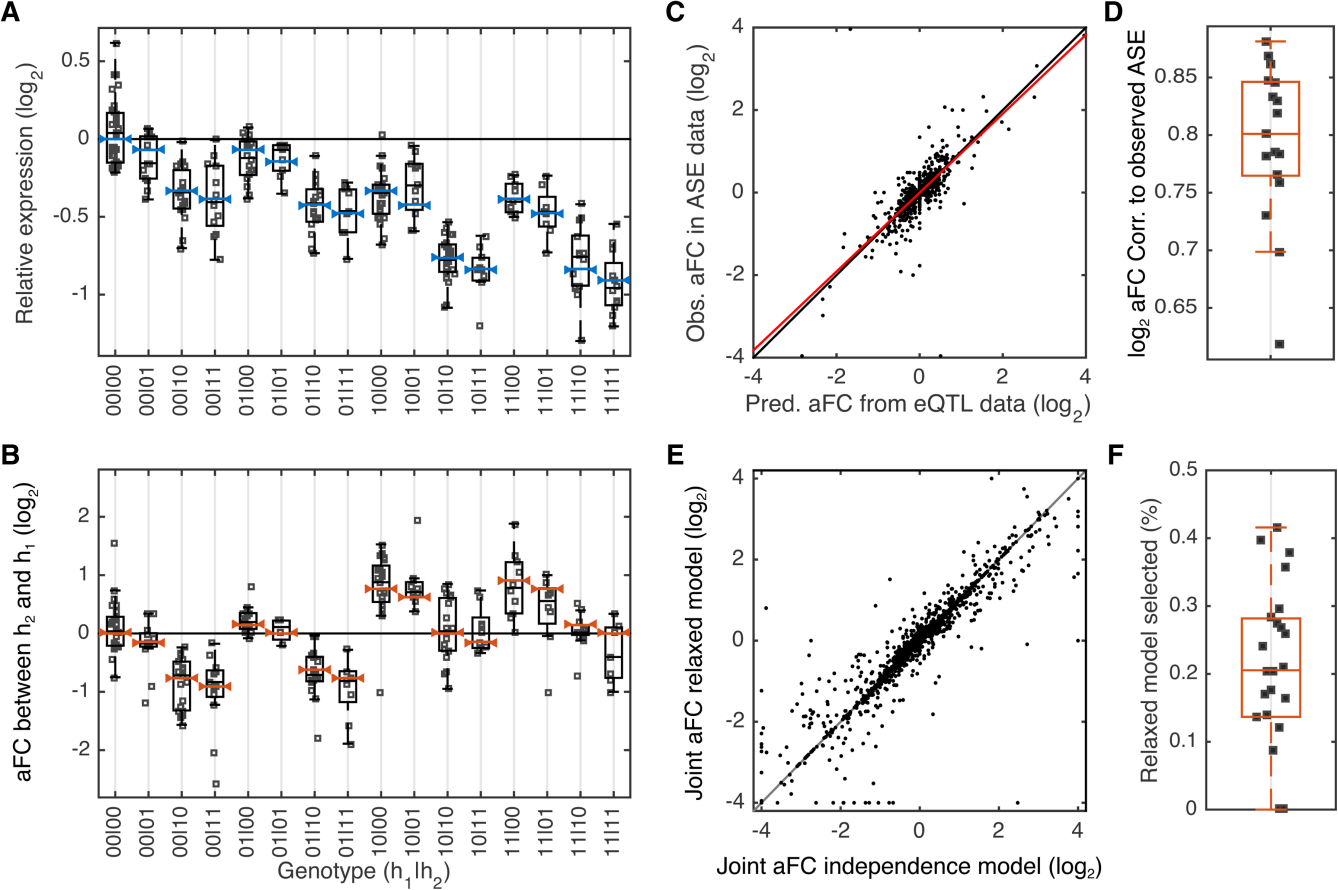
Joint analysis of aFCs for GTEx eGenes with two eQTLs. (*A*) An example of relative expression of eGene *ZC3H3* and the model fits for different genotype groups of its two eQTLs (eVariant1: Chr 8: 144633728 A/G; eVariant2: Chr 8: 144556836 G/A) in GTEx adipose subcutaneous. The effect size of the first and the second eQTLs are −0.77 and −0.14 as measured by log_2_ aFC. Each dot represents observed expression in one individual, scaled relative to the expression at all-reference genotype. The blue bars show model fits from the two-eQTL model based on regulatory independence assumption. Reference and alternative alleles are denoted by 0 and 1, respectively, and haplotypes are separated by “|” sign (e.g., 10|11 corresponds to the cases that one haplotype carries alternative and reference alleles of eVariant1 and eVariant2, respectively, and the other haplotype carries the alternative allele of both eVariants). (*B*) Expression of the second haplotype relative to the first haplotype, observed in ASE data. The red bars show expected haplotype expression ratios based on the model in panel *A*, learned on the eQTL data. (*C*) aFC between two haplotypes as predicted from eQTL data compared with median aFC observed in ASE data for all eGenes with two eQTLs in adipose subcutaneous. Each dot represents one randomly selected genotype for one eGene. Red line indicates the robust linear fit (*y* = 0.9*x* + 0.002). (*D*) Predicted and observed median aFC for all eGenes with two eQTLs calculated from eQTL and ASE data, respectively, in each tissue with more than 200 eGenes with two eQTLs. (*E*) *cis*-Regulatory effect size associated with co-occurrence of the alternative alleles of the two eQTLs, as predicted under regulatory independence model or learned using the relaxed model. (*F*) Percentage of the two eQTLs that are not well described using the independent regulatory assumption across all tissues with more than 200 eGenes with two eQTLs.

Iterative greedy procedures have been utilized to find multiple distinct eQTLs signals for each eGene in the GTEx data (Methods) (The GTEx Consortium 2017). We used GTEx eGenes with two distinct eQTLs to demonstrate how the aFC calculation can be extended to gain mechanistic insight into more complex eQTL patterns. The expression model in Equation 7 written for two biallelic eVariants was used in a nonlinear regression to simultaneously estimate the aFC associated with both eQTLs (Fig. 6A; Supplemental Table S1). These estimates were used to predict the relative expression of the two haplotypes between the 16 possible haplotypic combinations. We found that the predicted values from eQTL data correlate well with the observed values in ASE data across the genotypes (median *r* = 0.81) (Fig. 6B–D). Our generalized expression model inherently accounts for specific arrangement of the alleles for the two eVariants on haplotypes (e_11,00_ > e_10,01_) (Supplemental Fig. S6A). Specifically, according to the model, in individuals that are heterozygous for both eQTLs, the eGene is expected to have higher expression when the two higher expressed alleles occur on the same haplotype (e_HH,LL_ > e_HL,LH_) (Supplemental Fig. S6B). By using eGenes with two eQTLs in adipose subcutaneous, we found that this predicted effect of haplotype arrangement on eGene expression is consistent with the observed expression data (*r* = 0.43, *P* = 10^−25^) (Supplemental Fig. S6C–F).

Next, we considered the modeling assumption that the two eVariants act independently. Under this assumption, regulatory activity of the alleles from the first eQTL does not depend on the genotype at the second eQTL site and vice versa; therefore, the change in expression of the haplotype carrying the alternative allele for both eVariants is the multiplication of the two aFCs for each individual eVariant (${\text{e}}_{11}={\text{e}}_{00}\delta_{1,0}^{v1}\delta_{1,0}^{v2}$; Equation 5). In order to analyze how well the data are described assuming the independence of the two eVariants, we relaxed this assumption to allow for interactions by defining the joint genotype of the two eVariants as the genotype of a hypothetical variant with four possible alleles. We used Equation 7 written for one four-allelic eVariant to separately estimate the aFC associated with each of the two eVariants, as well as the aFC of their co-occurrence. We found that the estimates from the two models generally agree very well (Fig. 6C). We used the Bayesian information criterion (BIC) within a bootstrapping scheme to decide if relaxing the regulatory independence assumption provides a significantly better description of the data. This could be a sign of biological mechanisms such as epistasis or dosage compensation, as well as confounding factors such as linkage disequilibrium or expression quantification artifacts (Brown et al. 2014; Hemani et al. 2014; Wood et al. 2014; Fish et al. 2016). After accounting for the increased model complexity and uncertainty associated with sampling distribution, we found that only in 0.2% (range across tissues [0, 0.42]) of the two eQTLs for the same gene in GTEx data does the regulatory independence model fail to provide an adequate fit (Fig. 6D; Supplemental Fig. S7; Supplemental Table S1). This finding suggests that distinct eQTL signals identified using the iterative approach are largely driven by independent regulatory mechanisms. We note that the popular iterative discovery approach may be biased toward better discovery of independently acting eQTLs, and future work applying our method to distinct eQTLs discovered by other methods will be required to fully quantify the joint effects of *cis*-regulatory variants in human populations.

## Discussion

Despite over a decade of eQTL analysis and its increasingly widespread use in functional and medical genetics, eQTL effect size has lacked a consensus definition that is founded upon molecular interpretation of *cis*-regulation and is analytically convenient for broad use. Here, we described log aFC, a generalizable measure of *cis*-regulatory effect size that captures the mechanistic regulation of haplotype expression in *cis*. Log aFC is consistent across expression levels and allele frequencies and holds mathematically convenient properties that facilitate its application for downstream analysis. We show that aFC model for a single biallelic eQTL SNP is analytically equivalent to linear regression under the additive noise assumption, and therefore, it can be used to obtain effect sizes for eQTLs discovered with standard eQTL calling methods, as well as confidence intervals for aFC estimates that are consistent with eQTL significance. In addition to the aFC that captures the molecular effect, the proportion of expression variation explained by an eQTL in population data remains useful as a complementary measure valuable for describing population-level effect of an eQTL. aFC provides uniform estimates from both allelic expression and *cis*-eQTL data, and replication of *cis*-eQTLs using orthologous ASE data from the same samples can complement classical replication with an independent sample. Furthermore, estimating aFC from ASE and from eQTL data can prove useful in other scenarios. For instance, ASE-based estimation allows for exploring effects of *cis*-regulatory variation in single individuals, while this is not possible using total expression data (Kukurba et al. 2014; Rivas et al. 2015; The GTEx Consortium 2015).

While the correlation between effect sizes estimated from ASE and eQTL data is high, this is still likely an underestimate and could be improved by using methods that produce more accurate measures of haplotypic expression (Castel et al. 2016). The two alternative aFC calculation methods provided use untransformed and log-transformed eQTL data to account for additive and multiplicative noise, respectively. We showed that the estimates that utilize log-transformed data are generally better. However, both methods perform well, and the preferred noise model can vary depending on the expression measurement platform and upstream preprocessing pipelines that have been utilized. We benchmarked aFC for RNA sequencing data, the most popular platform for expression level quantification that provides both ASE and eQTL data, but aFC is a general measure, and the presented methods provided for eQTL data can be directly applied to data from other quantification platforms such as microarray and qPCR. Systematic extension of aFC-based model of *cis*-regulation to multiple alleles and multiple eQTLs, as demonstrated for the eGenes with two eQTLs in GTEx, allows investigating more complex problems while maintaining mechanistic interpretability of the results. By using the extended model, we showed that the haplotypic arrangement of the alleles of two distinct eQTLs affecting the same gene is important for accurate estimation of gene expression. We also found that the overwhelming majority of distinct eQTLs for the same gene found using the popular iterative eQTL discovery approach are likely to be driven by independent regulatory mechanisms, although future work is needed to study whether this applies to *cis*-regulatory variants in general. Finally, we introduced practical guidelines and a tool for estimating aFC from real data and provided a catalog of *cis*-eQTL effect sizes across all GTEx tissues as a resource for future studies.

A biologically interpretable and well-defined eQTL effect size estimate enables diverse downstream applications. By using the GTEx data set (The GTEx Consortium 2017), we have investigated differences in effect sizes among eGene types, eVariant annotations, and eQTL tissue specificity. Even though aFC itself is unbiased with respect to allele frequency and expression level, we showed here that it is essential for all downstream analyses to take into account factors that indirectly confound the effect size distribution via differences in eQTL discovery power. eQTL effect size quantification will be valuable for making quantitative comparisons between effects on gene expression and other phenotypes at the cellular and physiological level. Indeed, our method is generally applicable to estimating effect size of *cis*-regulatory variants affecting other cellular traits such as methylation, chromatin state, and protein levels as long as the general *cis*-regulatory assumption underlying the model remains realistic. Furthermore, due to the additive nature of log aFC, the magnitude of difference between two effect sizes is a readily interpretable statistic. This feature makes log aFC a useful tool for future characterization of variation in eQTL activity across cellular or environmental contexts. For disease-associated eQTLs, understanding the relationship between the quantitative expression effect in the cells and disease risk will be important for understanding molecular mediators of disease risk. Finally, the recent development of experimental approaches such as MPRA (Tewhey et al. 2016; Ulirsch et al. 2016), STARR-seq (Arnold et al. 2013; Vockley et al. 2015), and CRISPR genome editing assays (Canver et al. 2015; Wright and Sanjana 2016) has created demand for translating summary statistics of eQTL mapping to quantifications that are interpretable as reflecting molecular events in the cell. Our biologically interpretable estimates of *cis*-eQTL effect sizes from population data can be directly compared with in vitro quantification of regulatory variant effects.

## Methods

### Estimating *cis*-regulatory effect of an eVariant from allelic expression data

Standard RNA sequencing reads can be used to measure the expression of each of the two gene copies, via allelic counts in individuals carrying a heterozygous SNP (aeSNP) inside the transcribed region of the gene (Castel et al. 2015). Allelic counts provide measurement of the true allelic expression *e*_0_ and *e*_1_ from Equation 1 in a given sample on a relative scale (Supplemental Fig. S1). Since both measurements are drawn from the same sample, they share the same basal expression (*e*_*B*_ in Equation 1), and thus in absence of noise, the ratio between the two allelic counts directly reflects the effect of the *cis*-regulatory variant. Given allelic expression data from a set *N* of individuals heterozygous for an eVariant of interest, the aFC can therefore be robustly estimated as

(8) $$\delta_{1,0}=\underset{n=1\ldots N}{\text{median}}\frac{c_{1,n}}{c_{0,n}},$$

where c_0,n_ and *c*_1,n_ are the allelic counts in the *n*th individual for haplotype carrying reference and alternative allele for the *cis*-regulatory variant, respectively. Here we assume phasing between the regulatory alleles and the aeSNP alleles are known. In cases when phasing information is not available, the magnitude of the regulatory effect size can be calculated as

(9) $$d_{1,0}=\left|\log_{2}\delta_{1,0}\right|=\underset{n=1\ldots N}{\mathrm{median}}\left|\mathrm{lo}g_{2}\frac{c_{1,n}}{c_{0,n}}\right|.$$

However, this estimate without phasing information is more sensitive to noise and will systematically overestimate the effect size, particularly in cases where the true effect size is small in magnitude and the variation in allelic counts is dominated by measurement noise.

### Estimating *cis*-regulatory effect of an eVariant from gene expression data

#### Gene expression is linear with the number of alternative alleles for biallelic eVariants

By using Equation 4, we can derive gene expression in an individual as function of the number of alternative alleles, *t*:

(10) $$e(t)=[(2-t)+t\delta_{1,0}]e_{0},$$

where *t* is 0, 1, and 2 for individuals homozygous for reference allele, heterozygous, and homozygous for alternative allele, respectively. This equation can be written as

(11) $$e=\;b_{1}t+b_{0},$$

where

(12a) $$b_{0}=2e_{0},$$

(12b) $$b_{1}=e_{0}(\delta_{1,0}-1),$$

showing that total gene expression under a *cis*-regulatory model is linear for the number of alternative alleles of the variant (Fig. 1C). For estimating the aFC from expression data, we consider two cases of noise distribution: additive and multiplicative noise.

#### Estimating aFC from eQTL data with additive noise

Under an additive noise model, the measured gene expression in the *n*th individual, *y*_*n*_, is the true expression, *e*(*t*), plus a normally distributed noise, ε_n_, with zero mean and unknown variance. By using *e*(*t*) from Equation 10,

(13) $$y_{n}=[(2-t_{n})+t_{n}\delta_{1,0}]e_{0}+\varepsilon_{n},$$

where *t*_*n*_ is the number of alternative allele in the individual. Similar to Equation 10, Equation 13 can be written in linear form:

(14) $$y_{n}=b_{1}t_{n}+b_{0}+\varepsilon_{n}.$$

Maximum likelihood estimates for *b*_0_ and *b*_1_ can be derived efficiently using ordinary least squares, and solving Equations 12a and 12b, for δ_1,0_, the aFC is

(15) $$\delta_{1,0}=\frac{2b_{1}}{b_{0}}+1.$$

#### Estimating aFC from eQTL data with multiplicative noise

Assuming a multiplicative noise model, the measured gene expression in the *n*th individual, *y*_*n*_, is the true expression, *e*(*t*), multiplied by a noise, ε_n_, such that log ε_n_ is normally distributed with zero mean and unknown variance. Substituting *e*(*t*) from Equation 10 again,

(16) $$y_{n}=[(2-t_{n})+t_{n}\delta_{1,0}]e_{0}\varepsilon_{n}.$$

Due to the multiplicative noise, this equation can no longer be solved as a simple linear regression problem. Applying log transformation to both sides,

(17) $$z_{n}=\log_{2}y_{n}=\log_{2}[(2-t_{n})+t_{n}\delta_{1,0}]+\;\log_{2}e_{0}+\log_{2}\varepsilon_{n}.$$

The noise is captured by log_2_ ε_n_, which is additive and normally distributed, but the right side of the equation is no longer linear for the number of alternative alleles (Fig. 1D). By using nonlinear least squares optimization, Equation 17 can be solved to derive maximum likelihood estimates for the effect size δ_1,0_ directly.

#### Efficient approximation of aFC from eQTL data with multiplicative noise

Nonlinear least squares optimization needed for solving regression problem in Equation 17 is done using iterative numerical optimization that is a relatively slow procedure and not always straightforward to implement. In order to improve efficiency, we use four simplified linear models to derive four candidate estimates of the effect size and choose the one that provides the highest likelihood of the data. First, we derive three estimates of the regulatory effect size using the ratio of the expressions between each of the two genotypes:

(18a) $$\delta_{1,0}^{*1}=\frac{m_{2}}{m_{0}},$$

(18b) $$\delta_{1,0}^{*2}=\frac{1}{2\frac{m_{1}}{m_{2}}-1},$$

(18c) $$\delta_{1,0}^{*3}=2\frac{m_{1}}{m_{0}}-1,$$

where *m*_0_, *m*_1_, and *m*_2_ are the geometric means of expression in the samples homozygous for reference allele (*t*_*n*_ = 0), heterozygous (*t*_*n*_ = 1), and homozygous for the alternative allele (*t*_*n*_ = 2), respectively (see Supplemental Methods). When the *cis*-regulatory effect size approaches zero, the log-transformed gene expression is linear with number of alternatives alleles (See Supplemental Methods). Therefore, the nonlinear model in Equation 17 can be well approximated with linear regression in cases where the effect size is small (log _2_δ_1,0_ → 0). We regress log-transformed expressions on the genotype,

$$z_{n}=c_{1}t_{n}+c_{0}+\log_{2}\varepsilon_{n},$$

and calculate the fourth effect-size estimate as (see Supplemental Methods)

(20) $$\delta_{1,0}^{*4}=2^{2c_{1}}.$$

Residual of the fit, *r*_n_, in the *n*th sample for a given effect size estimate, δ_1,0_^*k^, is

(21) $$r_{n}(k)=z_{n}-\log_{2}[(2-t_{n})+t_{n}\delta_{1,0}^{*k}].$$

The estimate with lowest variance of the residuals among the four candidates is reported:

(22) $$\delta_{1,0}=\delta_{1,0}^{*I},\;I=\underset{i\in 1\ldots 4}{\text{argmin}}\text{V[}r(i)].$$

### Simulation experiment

The simulated data set includes 200 individuals and 10,000 eGenes each associated to exactly one eQTL. Each eQTL has two alleles; frequency of the reference allele, *f*_0_, was drawn from a uniform distribution for each eQTL (*f*_*0*_ ∼ *uniform*[0,1]). The eQTL genotype in each individual was decided using two Bernoulli trials. Reference and alternative alleles induce expressions *e*_0_ and *e*_1_ = δ_1,0_ *e*_0_ in the eGene in *cis*, respectively (Equations 1, 2). The expression *e*_0_ is generated for each eGene randomly across four orders of magnitude (log_10_ *e*_0_ ∼ *uniform*[0,4]). Similarly, the aFC, δ_1,0_, was assumed to be uniformly distributed in logarithmic scale (log_2_ δ_1,0_ ∼ *uniform*[−5,5]) across simulated eQTLs. In order to choose a realistic noise level, we used data from all eGenes associated with eQTLs in GTEx. For each eQTL genotype class, expression mean and variance of the associated eGene was calculated. As expected, gene expression was highly heteroskedastic with the mean–variance relationship resembling that of multiplicative noise by log-normal distribution (Supplemental Fig. S2). We used average within genotype standard deviation of log_10_-transformed gene expression to add log-normal noise in the simulation (log_10_ ε_n_ ∼ *norm*[0, σ = 0.17]; Equation 17).

### Estimating aFC for GTEx eQTLs

#### ASE-based estimates

Haplotypic counts were generated as described by The GTEx Consortium (2017). Briefly, allelic counts for each sample were generated from uniquely aligned RNA-seq reads for all heterozygous SNPs from OMNI Array imputed genotypes using the GATK ASEReadCounter tool (Castel et al. 2015). SNPs covered by less than eight reads, those that showed bias in mapping simulations (Panousis et al. 2014), those that had a UCSC 50-mer mappability lower than one, or those without evidence for heterozygosity (Castel et al. 2015) were excluded. The expression associated with each eQTL allele haplotype was obtained by summing up allelic counts within a gene using population phasing relative to the eQTL variant (eVariant) for each sample. All individuals that are heterozygous for the eVariant were used in Equation 8 to calculate eQTL effect size from haplotypic counts. Bias-corrected and accelerated bootstrap was applied to infer 95% confidence intervals for the aFC estimates (Efron 2012).

#### eQTL-based estimates

For eQTL data, expression counts were scaled for the total library size, and one pseudocount was added to smooth the normalized counts. Log-transformed expression data were corrected for confounding factors identified using PEER (Stegle et al. 2012) and the three top principal components of the genotype matrix uniformly for all three tested methods: linear, nonlinear, and nonlinear approximation. The correction was done in two steps: First, the log-transformed expression profile of the eGene in *n*th sample, *z*_n_, was modeled using linear regression:

(23) $$z_{n}=\mu +\alpha C_{n}+\beta_{t_{n}}+\varepsilon_{n},$$

where *C*_n_ is the *n*th column of the matrix *C*_*M×N*_ containing *M* confounding factors, and *t*_n_ ∈ {0, 1, 2} indicates the number of alternative alleles in the *n*th sample. All nonsignificant columns, for which the 95% confidence interval of the regression coefficient in α overlapped zero, were discarded from *C*. In the second step, the regression was repeated using the reduced covariate matrix, and corrected expression was derived as

(24) $$\hat{z}=z-\alpha C.$$

The corrected expression vector, $\hat{z}$, was used for effect size calculations. For direct estimation of aFC from Equation 17 (the nonlinear method, M2, in Figs. 3, 4), we used the Matlab generic nonlinear least square solver (*lsqnonlin*). The effect size estimates used in Figure 5, as well as those published on GTEx portal (http://gtexportal.org), were calculated using the nonlinear approximation method (M3), and the 95% confidence intervals for the aFC estimates were calculated using the bias-corrected and accelerated bootstrap (Efron 2012). The full data of the GTEx V6p release are available in dbGaP (study accession phs000424.v6.p1), and eQTL summary statistics, including the effect size estimates for the top eVariant–eGene pair per tissue, are available from the GTEx portal (http://gtexportal.org).

### Mapping multiple eQTL signals per eGene

Multiple distinct signals for a given expression phenotype were identified by forward stepwise regression followed by a backward selection step. The gene-level significance threshold was set to be the maximum beta-adjusted *P*-value (correcting for multiple testing across the variants) over all eGenes in a given tissue. At each iteration, we performed a scan for *cis*-eQTLs using FastQTL (Ongen et al. 2016), correcting for all previously discovered variants and all standard GTEx covariates. If the beta-adjusted *P*-value for the lead variant was not significant at the gene-level threshold, the forward stage was complete and the procedure moved on to the backward stage. If this *P*-value was significant, the lead variant was added to the list of discovered *cis*-eQTLs as a distinct signal and the forward step moves on to the next iteration. The backward stage consisted of testing each variant separately, controlling for all other discovered variants. To do this, for an eGene with *n* eVariants, we ran *n cis* scans (in effect *n* − 1 *cis* scans, as one replicates the final stage of the forward analysis). For each *cis* scan, we control for all covariates and all but one of the discovered eVariants (the one dropped is the genetic signal that is being tested, conditioned on the full model). If no variant was significant at the gene-level threshold, the variant in question was dropped, otherwise the lead variant from this scan, which controls for all other signals found in the forward stage, was chosen as the variant that represents the signal best in the full model.

### Joint analysis of two eQTLs

#### Regulatory independent model

Let us assume two biallelic eVariants, *v*_1_ and *v*_2_, regulating expression of the same eGene in *cis* (Supplemental Fig. S6A). This is a special case of Equations 5 through 7 where *N* = 2 and *m*_1_ = *m*_2_ = 2. Under the independence assumption, the regulatory effect of each eVariant allele on the expression of the carrying haplotype does not depend on the present allele for the other eVariant, and therefore, the expression of a haplotype carrying alleles *i*_1_ and *i*_2_ for the two eVariants is

(25) $$e_{i_{1}i_{2}}=e_{0}\delta_{i_{1},0}^{v1}\delta_{i_{2},0}^{v2},$$

where indices *i*_1_, *i*_2_ ∈ {0, 1} indicate reference (zero) and the alternative allele (one); $\delta_{i_{1},0}^{v1}$, and $\delta_{i_{2},0}^{v2}$ are the aFCs associated with the present alleles relative to the reference allele for *v*_1_ and *v*_2_, respectively; and *e*_0_ is the expression of a haplotype carrying reference allele for both eVariants. Under this model, the log ratio between the expressions of the two haplotypes is

(26) $${\text{s}}_{i_{1}i_{2},j_{1}j_{2}}=\log_{2}\frac{e_{i_{1}i_{2}}}{e_{\;j_{1}j_{2}}},$$

where indices *i*_1_, *i*_2_ ∈ {0, 1} and *j*_1_, *j*_2_ ∈ {0, 1} indicate the present alleles on the first and second haplotype, respectively. From definition of Afc,

(27) $$\delta_{i,0}=\delta_{i,j}\delta_{\;j,0};$$

thus after substituting haplotypic expressions from Equation 25 in Equation 26, the log ratio between the expressions of the two haplotypes is

(28) $$s_{i_{1}i_{2},j_{1}j_{2}}=\log_{2}\left(\delta_{i_{1},j_{1}}^{v1}\delta_{i_{2},j_{2}}^{v2}\right)=s_{i_{1},j_{1}}^{v1}+s_{i_{2},j_{2}}^{v2}.$$

This equation presents the expected log aFC for a given genotype. Therefore, under the regulatory independence model, the joint effect of the two alternative alleles is sum of their individual effects:

(29) $$s_{11,00}=s_{1,0}^{v1}+s_{1,0}^{v2}.$$

Under the *cis*-regulatory model, total expression of the eGene for each genotype is the some of the individual haplotype expressions:

(30) $$e_{i_{1}i_{2},j_{1}j_{2}}=e_{i_{1}i_{2}}+e_{\;j_{1}j_{2}}.$$

Substituting haplotypic expressions from Equation 25, we can use measured expression profiles of genotyped individuals to estimate aFC associated with the two eVariants. The observed expression value for the eGene in the *n*th sample after log transformation is

(31) $$\begin{array}{rl} z_{i_{n,1}i_{n,2},j_{n,1}j_{n,2}} & =\log e_{0}+\log\left(\delta_{i_{n,1},0}^{v1}\delta_{i_{n,2},0}^{v2}+\delta_{\;j_{n,1},0}^{v1}\delta_{\;j_{n,2},0}^{v2}\right)\\ & \;+\alpha C_{n}+\varepsilon_{n}, \end{array}$$

where indices *i*_*n*,1_, *i*_*n*,2_, *j*_*n*,1_, *j*_*n*,2_ ∈ {0, 1} indicate the present alleles, and *C*_n_ is the provided column vector of the confounding factors for the sample. The nonlinear regression problem can be solved to estimate reference expression *e*_0_, individual aFC effects δ_1,0_^*v*1^, δ_1,0_^*v*2^, and the cofactor weight vector α (by definition δ_0,0_^*v*1^ and δ_0,0_^*v*2^ are each equal to 1).

In order to estimate aFCs for eGenes with two eQTLs in GTEx data, we used PEER (Stegle et al. 2012) and top three principal components of the genotype matrix as the confounding factors in matrix *C*. Generic nonlinear least square optimizer in Matlab (*lsqnonlin*) was used to derive parameter estimates for the Equation 26 regression problem. Confidence intervals of the parameters were derived using the *t*-statistic estimated via Jacobean matrix calculated at the optimal function values (Matlab function: *nlparci*). Predicted aFCs for regulatory independence model presented in Figure 6, B through E, and Supplemental Figure S7C (blue bars) were derived using Equation 28. The prediction of haplotype arrangement effects in Supplemental Figure S6 were derived using Equations 30 and 31.

#### Relaxed model

In this model, we relax the regulatory independence assumption, allowing the regulatory effect associated with co-occurrence of the two alternative alleles to be potentially different from sum of their individual effects. In contrast to Equation 25, haplotype expression is

(32) $$e_{i_{1}i_{2}}=e_{0}\delta_{i_{1}i_{2},00},$$

where $\delta_{i_{1}i_{2},00}$ is the aFC associated to copresence of the alleles *i*_1_ and *i*_2_ of the eVariants *v*_1_ and *v*_2_ compared with a haplotype carrying reference allele for both eVariants. This model is equivalent to a special case of models in Equations 5 through 7, where *N* = 1 and *m*_1_ = 4. From the aFC definition,

(33) $$\delta_{i_{1}i_{2},00}=\delta_{i_{1}i_{2},j_{1}j_{2}}\delta_{\;j_{1}j_{2},00},$$

and the log ratio between the expressions of the two haplotypes is

(34) $$s_{i_{1}i_{2},j_{1}j_{2}}=\log_{2}\delta_{i_{1}i_{2},j_{1}j_{2}}=s_{i_{1}i_{2},00}-s_{\;j_{1}j_{2},00}.$$

The total expression is the sum of the individual haplotypic expressions (Equation 30); thus, the observed expression value for the eGene in the *n*th sample under the relaxed regulatory model after log transformation is

(35) $$z_{i_{n,1}i_{n,2},j_{n,1}j_{n,2}}=\log e_{0}+\log\left(\delta_{i_{n,1}i_{n,2},00}+\delta_{\;j_{n,1}j_{n,2},00}\right)+\alpha C_{n}+\varepsilon_{n},$$

where indices *i*_n,1_, *i*_n,2_, *j*_n,1_, *j*_n,2_ indicate the present alleles and *C*_n_ the covariates as described in Equation 31. The nonlinear regression problem can be solved for reference expression *e*_0_, joint aFC effects δ_10,00_, δ_01,00_, δ_11,00_, and the cofactor weight vector α (by definition δ_00,00_ is equal to one).

To estimate aFCs in GTEx data, regression parameters and their confidence intervals were estimated as described for the regulatory independence model. Predicted aFCs for the relaxed model presented in Figure 6E and Supplemental Figure S7C (red bars) were derived using Equation 34.

#### Model comparison

In order to compare the two models of *cis*-regulation, the independence and the relaxed model, we calculated total data likelihood for each of the models under the log-normality assumption:

(36) $$L\left(z|M\right)=\overset{N}{\underset{n=1}{\prod }}\frac{1}{\sigma \sqrt{2\pi }}e^{-\frac{r_{n}^{2}}{2\sigma^{2}}},$$

where *z* is the vector of *N* samples, *r*_n_ is the fit residual at the *n*th sample using the model considered M, and σ is the standard deviation of the fit residuals. Bayesian information criterion (BIC) for each of two models was calculated:

(37) BIC(M)=−2logL(z|M)+λlog⁡N,

where λ, the number of parameters in each model, is the number of cofactor coefficients plus three and plus four for the regulatory independence and the relaxed model, respectively. We used bias-corrected and accelerated bootstrap (Efron 2012) to estimate confidence intervals for ΔBIC = BIC(Relaxed model) − BIC(Independence model) in cases where ΔBIC is negative. The relaxed model was selected in cases where the upper bound for the 95% confidence interval for ΔBIC fell below zero, and for the rest of the cases, the independence model that has fewer parameters was deemed adequate. The calculated aFCs for all eGenes in GTEx with two associated eQTLs are provided in Supplemental Table S1.

### Software availability

Software for calculating aFC from standard eQTL data is provided in Supplemental Software S1 and is available online on GitHub (https://github.com/secastel/aFC).

## Supplementary Material

Supplemental Material
