# Supplementary material for: Quantifying the regulatory effect size of cis-acting genetic variation using allelic fold change
Source: Genome Res. 2017 Nov;27(11):1872–84. doi: 10.1101/gr.216747.116 (PMC5668944; doi:10.1101/gr.216747.116)
Supplement: Supplemental Material [file supp_27_11_1872__index.html]

Quantifying the regulatory effect size of cis-acting genetic variation using allelic fold change — Supplemental Material 

# Quantifying the regulatory effect size of *cis*-acting genetic variation using allelic fold change

## Supplemental Material

- Supplemental\_Material.pdf
- Supplemental\_Table\_S1.xlsx
- Supplemental\_Software\_S1.zip
