# Supplementary material for: Quantifying the regulatory effect size of cis-acting genetic variation using allelic fold change
Source: Genome Res. 2017 Nov;27(11):1872–84. doi: 10.1101/gr.216747.116 (PMC5668944; doi:10.1101/gr.216747.116)
Supplement: Supplemental Material [file supp_gr.216747.116_Supplemental_Material.pdf]

Quantifying the regulatory effect size of *cis*-acting genetic variation using allelic fold change

Supplemental material

Table of Contents

Supplemental methods **2**
Derivations and proofs *2*
Supplemental Figures **7**
Supplemental Table Legend **12**

Supplemental methods

Derivations and proofs

1. **Proof: Log-transformed eGene expression is linear in the number of alternative alleles as the *cis*-regulatory effect size approaches zero.**

Let α_1 and α_2 be the slope of the line connecting eGene expressions from reference homozygous to heterozygous, and from heterozygous to homozygous alternative genotype, respectively, in the piecewise linear model of log-transformed eQTL data (**Figure 1C**):

$$\begin{aligned} \alpha_1 &= \log e_{1,0} - \log e_{0,0} \\ \alpha_2 &= \log e_{1,1} - \log e_{1,0} \end{aligned} \tag{S1}$$

In a linear model α_1 is equal to α_2. Substituting the allelic expressions from the main text **Eq. 4**, the ratio between the two slopes for weak eQTLs is

$$\lim_{s_{1,0}\to 0} \frac{\alpha_1}{\alpha_2} = \lim_{s_{1,0}\to 0} \frac{\log(2^{s_{1,0}} + 1) - \log 2}{\log 2 + \log 2^{s_{1,0}} - \log(2^{s_{1,0}} + 1)} \tag{S2}$$

where, $s_{1,0} = \log_2 \delta_{1,0}$ is the eQTL effect size. Since, the limit value for both nominator and the denominator is 0, we apply L'Hôpital's rule

$$\lim_{s_{1,0}\to 0} \frac{\alpha_1}{\alpha_2} = \lim_{s_{1,0}\to 0} \frac{\alpha_1{}'}{\alpha_2{}'} = \lim_{s_{1,0}\to 0} \frac{\frac{1}{2^{s_{1,0}}+1}}{\frac{1}{2^{s_{1,0}}} - \frac{1}{2^{s_{1,0}}+1}} = \frac{\frac{1}{1+1}}{\frac{1}{1} - \frac{1}{1+1}} = 1 \tag{S3}$$

Thus, the two slopes, α_1 and α_2 are equal in weak eQTLs as $s_{1,0} \to 0$.

2. **Derivations: Approximate nonlinear model for aFC estimation**

Let us assume t_n is the number of alternative allele in n^{th} sample, and m_0, m_1, and m_2 are the geometric means of expression in the samples homozygous for reference allele ($t_n = 0$), heterozygous ($t_n = 1$), and homozygous for the alternative allele ($t_n = 2$) respectively. First, we use the expression ratio between each of the two genotype classes to estimate aFC. From **Eq. 17**, the expected log-transformed expression at each eQTL genotype class is

$$\mathrm{E}[z_n | t_n = 0] = \log_2 e_0 + 1 \tag{S4a}$$
$$\mathrm{E}[z_n | t_n = 1] = \log_2 e_0 + \log_2(\delta_{1,0} + 1) \tag{S4b}$$
$$\mathrm{E}[z_n | t_n = 2] = \log_2 e_0 + \log_2 \delta_{1,0} + 1 \tag{S4c}$$

Using **Eqs. S4a**, and **S4c**, the $\log_2$ aFC is

$$\mathrm{E}[z_n | t_n = 2] - \mathrm{E}[z_n | t_n = 0] = \log_2 \delta_{1,0} \tag{S5}$$

Substituting observed geometric means $m_t = 2^{\mathrm{E}[z_n|t_n=t]}$, and exponentiating both sides of the equation, the aFC is

$$\delta_{1,0} = \frac{m_2}{m_0} \tag{S6}$$

Next, we use **Eqs. S4b**, and **S4c**:

$$\mathrm{E}[z_n|t_n = 2] - \mathrm{E}[z_n|t_n = 1] = \log_2 \delta_{1,0} + 1 - \log_2(\delta_{1,0} + 1) \tag{S7}$$

Exponentiating the both sides we have

$$\frac{2^{\mathrm{E}[z_n|t_n=2]}}{2^{\mathrm{E}[z_n|t_n=1]}} = \frac{2\delta_{1,0}}{\delta_{1,0} + 1}$$

after substituting geometric means and rearranging the terms, the aFC is given:

$$\delta_{1,0} = \frac{1}{2\frac{m_1}{m_2} - 1} \tag{S8}$$

Using **Eqs. S4a**, and **S4b**

$$\mathrm{E}[z_n|t_n = 1] - \mathrm{E}[z_n|t_n = 0] = \log_2(\delta_{1,0} + 1) - 1 \tag{S9}$$

aFC can be similarly derived:

$$\delta_{1,0} = 2\frac{m_1}{m_0} - 1 \tag{S10}$$

As a fourth estimate, we use loglinear regression to derive another aFC estimate. This is an accurate model for weak eQTLs where the piece-wise linear eQTL model approaches linearity (see **Eqs. S1-3**). The regression line passes $\mathrm{E}[z_n|t_n = 0]$ at $t_n = 0$, and $\mathrm{E}[z_n|t_n = 2]$ at $t_n = 2$, therefore the slope, c_1, of the line is

$$c_1 = \frac{\mathrm{E}[z_n|t_n = 2] - \mathrm{E}[z_n|t_n = 0]}{2 - 0} = \frac{\log_2 \delta_{1,0}}{2} \tag{S11}$$

Thus aFC is given as

$$\delta_{1,0} = 2^{2c_1} \tag{S12}$$

It is worth noting that under the *cis*-regulatory model of **Eqs. 4a-c**, the expression in the heterozygous class is at least half of that of the higher expressed homozygous class, taking place when the weak allele is effectively zero expressed, thus:

$$-\infty \geq \mathrm{E}[z_n|t_n = 2] - \mathrm{E}[z_n|t_n = 1] \geq 1 \tag{S13a}$$
$$-\infty \geq \mathrm{E}[z_n|t_n = 0] - \mathrm{E}[z_n|t_n = 1] \geq 1 \tag{S13b}$$

In practice, the observed expression of the genotype classes, m_0, m_1, and m_2, can occasionally fall outside these boundaries due to noise or other confounding biological factors beyond the considered *cis*-regulatory model. Therefore, the ratios $\frac{m_1}{m_0}$, and $\frac{m_1}{m_2}$ in **Eqs. S8** and **S10** should be bound to be ≥0.5 to avoid negative aFC estimates.

3. Mathematical properties of log aFC

Recalling log aFC definition:

$$s_{i,j} = \log_2 \delta_{i,j}$$

$$= \log_2 e_1 - \log_2 e_0$$

$$= \log_2 k_i - \log_2 k_j$$

We show that the following statements are true:

S.I Zero log aFC indicates the absence of regulatory difference: $s_{i,i} = 0$

$$s_{i,i} = \log_2 k_i - \log_2 k_i = 0$$

S.II Choice of reference allele only affects the sign of log aFC: $s_{i,j} = -s_{j,i}$

$$\begin{aligned} s_{i,j} &= \log_2 k_i - \log_2 k_j \\ &= -(\log_2 k_j - \log_2 k_i) \\ &= -s_{j,i} \end{aligned}$$

S.III Log aFC is additive: $s_{i,k} = s_{i,j} + s_{j,k}$

$$\begin{aligned} s_{i,k} &= \log_2 k_i - \log_2 k_k \\ &= \log_2 k_i - \log_2 k_k + \log_2 k_j - \log_2 k_j \\ &= (\log_2 k_i - \log_2 k_j) + (\log_2 k_j - \log_2 k_k) \\ &= s_{i,j} + s_{j,k} \end{aligned}$$

S.IV aFC associated with joint effect of independent regulatory variants, $v1 \ldots v$N is sum of their individual log aFCs:

$$\mathbf{s}_{\langle i_1 \ldots i_n \ldots i_N \rangle, \langle j_1 \ldots j_n \ldots j_N \rangle} = \sum_{n=1}^{N} \mathbf{s}_{i_n j_n}^{vn}$$

where $\langle i_1 \ldots i_n \ldots i_N \rangle$ and $\langle j_1 \ldots j_n \ldots j_N \rangle$ are the set of present alleles on each of the haplotypes.

Assuming that variants affect gene expression independently, haplotype expression in **Eq. 1** in the main text can be written for *N* eVariants as

$$e_{\langle i_1 \ldots i_n \ldots i_N \rangle} = e_B \prod_{n=1}^{N} k_{i_n}^{v\mathrm{n}}$$

where $k_{i_n}^{v\mathrm{n}}$ denotes the regulatory effect on the eGene expression specific to allele i_n of the nth eVariant. Therefore, the joint aFC is

$$\mathrm{s}_{\langle i_1 \ldots i_n \ldots i_N \rangle, \langle j_1 \ldots j_n \ldots j_N \rangle} = \log_2 \frac{e_{\langle i_1 \ldots i_n \ldots i_N \rangle}}{e_{\langle j_1 \ldots j_n \ldots j_N \rangle}}$$

$$= \log_2 \frac{e_B \prod_{n=1}^{N} k_{i_n}^{v\mathrm{n}}}{e_B \prod_{n=1}^{N} k_{i_n}^{v\mathrm{n}}}$$

$$= \log_2 \prod_{n=1}^{N} \frac{k_{i_n}^{v\mathrm{n}}}{k_{j_n}^{v\mathrm{n}}}$$

$$= \sum_{n=1}^{N} \log_2 \frac{k_{i_n}^{v\mathrm{n}}}{k_{j_n}^{v\mathrm{n}}}$$

$$= \sum_{n=1}^{N} \mathrm{s}_{i_n, j_n}^{v\mathrm{n}}$$

S.V Absolute value of log aFC, $d_{i,j} = |s_{i,j}|$, is a pseudo-metric:

1. $d_{i,j} \geq 0$
2. $d_{i,i} = 0$
3. $d_{i,j} = d_{j,i}$
4. $d_{i,k} \leq d_{i,j} + d_{j,k}$

The first condition is met by definition and the second and third conditions are trivial considering the aFC properties **S.I** and **S.II** shown above. In order to demonstrate the truth of the fourth condition we consider two cases:

1) When $s_{i,j}$ and $s_{j,k}$ are both positive or both negative; in such cases due to additivity of log aFC (Statement **S.III**), $s_{i,k}$ will also have the same sign, and therefore, $d_{i,k} = d_{i,j} + d_{j,k}$ is trivial.

2) When $s_{i,j}$ and $s_{j,k}$ have different signs; Let us assume $s_{i,j} \geq 0$ and $s_{j,k} \leq 0$, from **S.III**:

$$\begin{aligned} s_{i,k} &= s_{i,j} + s_{j,k} \\ &= d_{i,j} - d_{j,k} \\ &\leq d_{i,j} + d_{j,k} \end{aligned}$$

Additionally,

$$\begin{aligned}-s_{i,k} &= -\left(s_{i,j} + s_{j,k}\right) \\ &= d_{j,k} - d_{i,j} \\ &\le d_{i,j} + d_{j,k} \\ &\quad \Rightarrow s_{i,k} \ge -\left(d_{i,j} + d_{j,k}\right)\end{aligned}$$

Combining the last two statements $d_{i,k} = |s_{i,k}| \le -d_{i,j} + d_{j,k}$. The opposite case where $s_{i,j} \le 0$ and $s_{j,k} \ge 0$, is the same.

Supplemental Figures

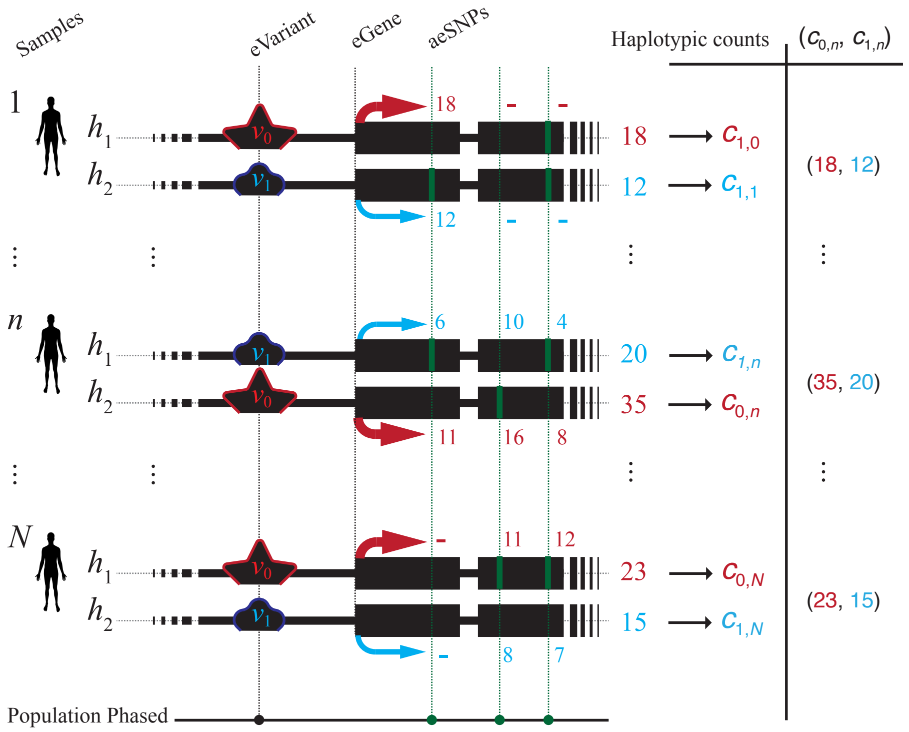

Supplemental Fig S1: Generating input data for calculating aFC from allelic expression data. Allelic expression data from individuals heterozygous for a given eQTL variant (eVariant) can be used to estimate the eQTL effect size. The two alleles of the eVariant and their associated expression values are shown in red (v_0; reference allele) and blue (v_1; alternative allele). In the above schematic there are three aeSNPs found in the eGene. Occurrence of the alternative allele for the aeSNPs is denoted by green bars. Allelic expression can be measured at each of the aeSNPs when the individual is heterozygous for the aeSNP. Phasing between the eQTL SNP and the aeSNPs is needed to enable aggregation of the allelic counts along the haplotypes that carry the reference ($c_{0,n}$), and the alternative allele of the eVariant $c_{1,n}$ in the nth individual. In our analyses, we use population reference based phasing of the GTEx genotype data.

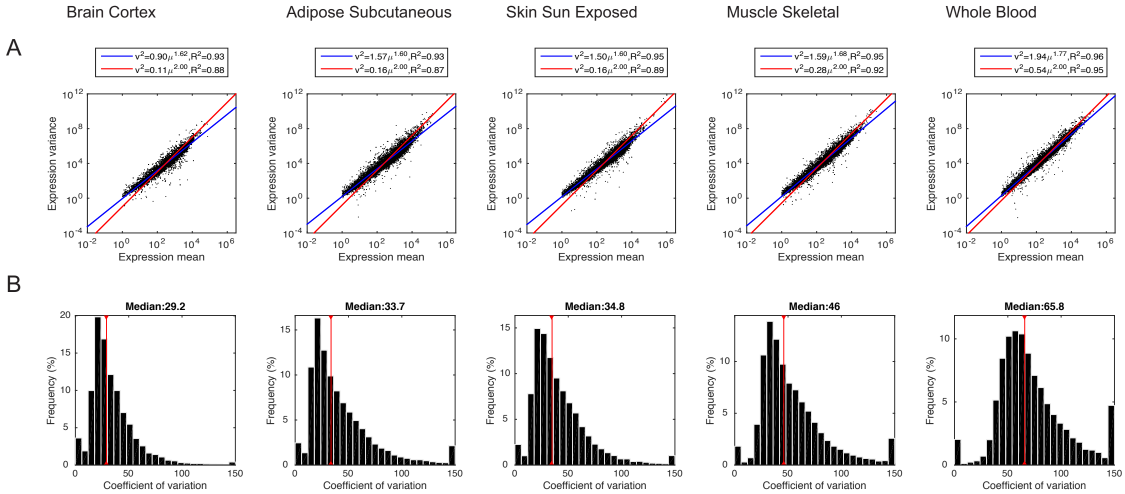

Supplemental Fig S2: Gene expression noise distribution in GTEx data. A) Mean and variance of eGene expression within genotype classes of the top eQTL for five example tissues in GTEx data. Each dot corresponds to data from one eGene within an eQTL genotype class. Red line indicates the best expected mean-variance dependence from lognormal distributed data, and blue lines shows the optimal linear regression line. This pattern shows that variance structure eQTL data is highly similar to lognormal distribution. B) Coefficient of variation, the ratio between the standard deviation and mean, for eGene expression within eQTL genotype classes for the same tissues.

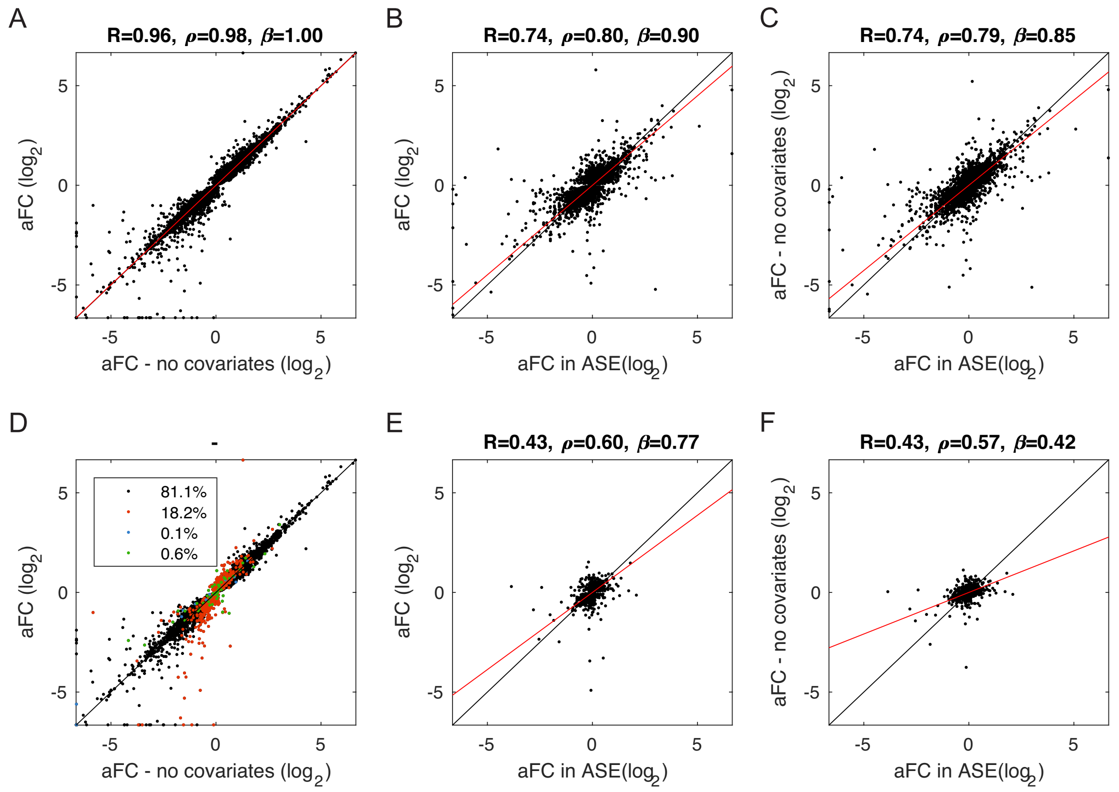

Supplemental Fig S3: The effect of correcting for confounding variation by PEER factors on aFC estimates. A) aFC values estimated on GTEx Adipose Subcutaneous eQTL data (n=8795) with and without correction for PEER factors as covariates. B-C) aFC estimates from eQTL data with (B) and without (C) correction compared to estimates using ASE data (n=5214). D) The effect of correcting for covariates on the confidence intervals of aFC estimates. Red denotes eQTLs where aFC estimates overlap zero if covariates are not included in the analysis (18.2%). Blue denotes the opposite cases where aFC overlaps zero when covariates are included (0.1%). Green denotes cases where the confidence intervals overlap zero regardless of covariate correction (0.6%). The aFC estimates with 95% confidence intervals that do not overlap zero correspond to eQTLs that are significant under 5% nominal p-value threshold. E-F) aFC estimates from eQTL data with (E) and without (F) correction compared to estimates using ASE data for the subset of eQTLs denoted in red on panel D (n=1250). R and ρ denote Pearson and Spearman correlation coefficients, respectively, β is the slope of the regression line from origin ($y=\beta x+\varepsilon$) that is shown in red.

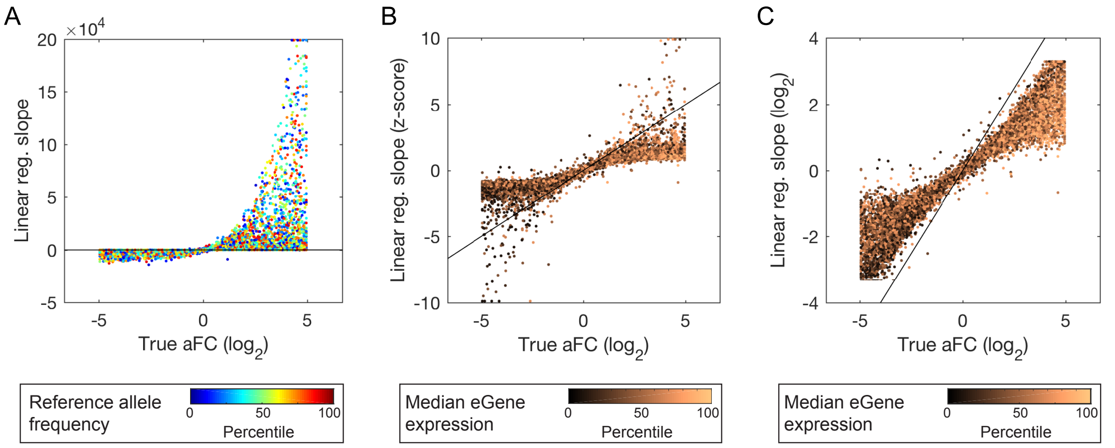

Supplemental Fig S4: Allelic fold change compared to linear regression slope. A-C) Slope of linear regression from 10,000 simulated eQTLs generated similar to data shown in **Figure 2**. The true aFC value is compared to regression slope from raw (A), z-scored (B), and $\log_2$ transformed data (C). The color code represents reference allele frequency (A), and median eGene expression (B-C); Alternative color-coding for the same plots are provided in **Figure 4**).

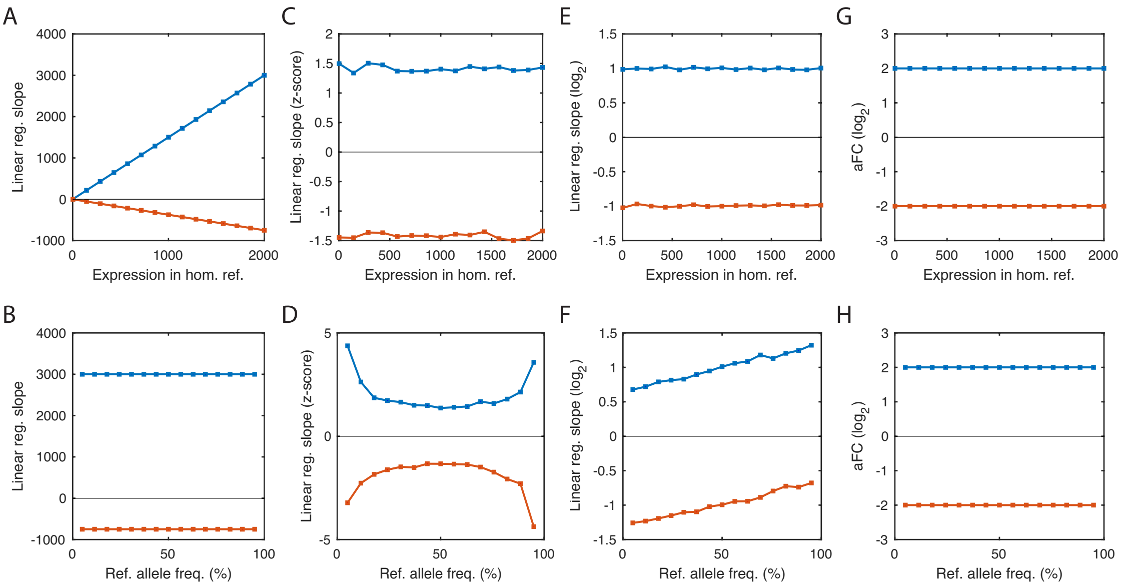

Supplemental Fig S5: Variation in linear regression slope driven by expression level and allele frequency. Shown are the estimated eQTL effect size values as the regression slope calculated on raw (A-B), z-scored (C-D), and $\log_2$ transformed expression data (E-F), as well as $\log_2$ aFC (G-H) for two simulated eQTLs. Blue points correspond to an eQTL in which the alternative allele is expressed four times higher compared to the reference, and the red points correspond to the opposite case where the reference allele is expressed four times the alternative. Upper and lower panels demonstrate the estimates derived from 15 repetitions of the simulation using a range of gene expression levels, and allele frequencies, respectively. Expression data was simulated for 200 individuals with Bernoulli sampled genotypes and no additional noise.

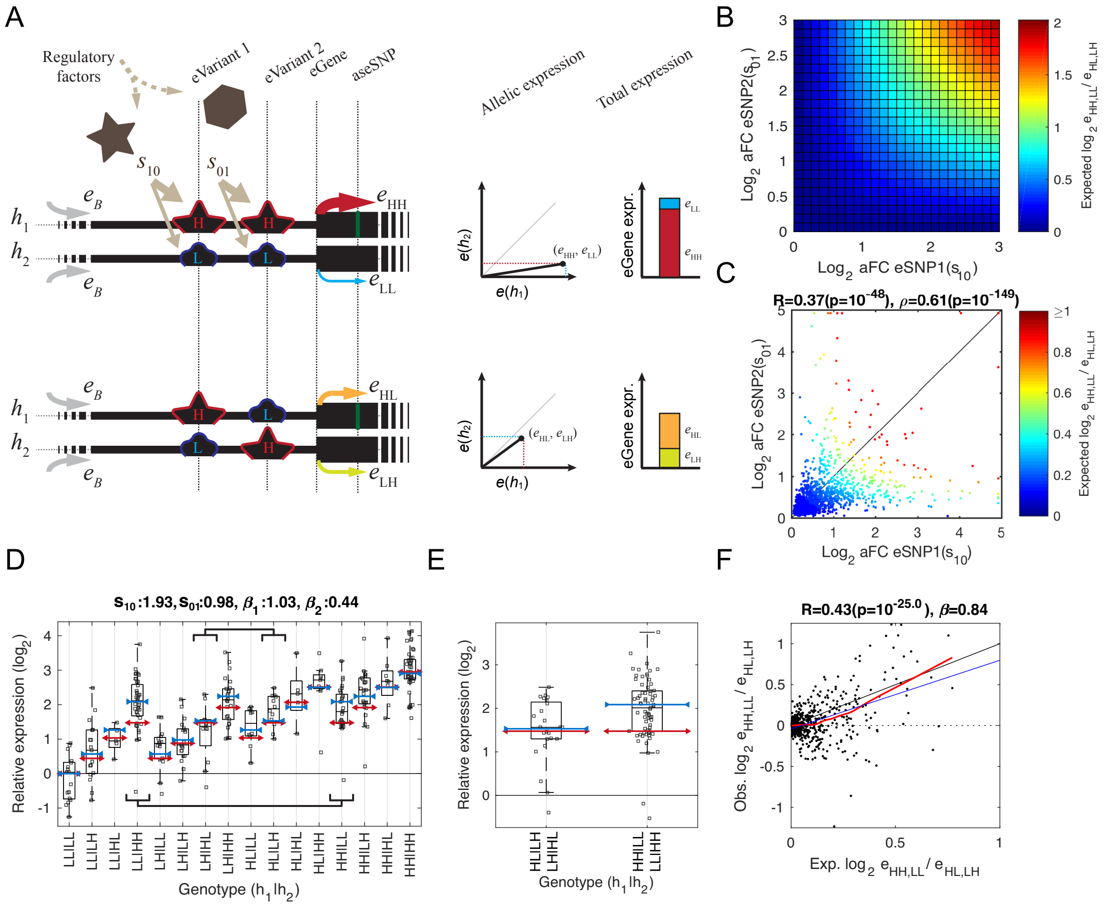

Supplemental Fig S6: Effect of haplotype arrangement on expression level. A) Schematic representation of two-variant *cis*-regulatory eQTL model in **Eq. 25-30** in individuals heterozygous for both eVariants. Higher and lower expressed alleles in each case are denoted by "H" and "L", respectively. Two possible haplotype arrangements are illustrated: "HH|LL" in which the higher expressed alleles are both on the same haplotype, and "HL|LH" where they are not. B-C) The expected effect of haplotype arrangement, $\log_2 e_{\langle HH\rangle,\langle LL\rangle}/e_{\langle HL\rangle,\langle LH\rangle}$, as a function of the effect sizes for the two eQTLs calculated over a continuous range of aFCs (B), and predicted for all eGenes with two distinct eQTL signals in GTEx Adipose Subcutaneous eQTL data (n=1472) (C). D) An example of relative expression of eGene *RP11-370B11.3* and the model fits for all genotype groups of its two eQTLs (eVariant1: Chr9:22767164 C/A and eVariant2: Chr9:22757714 A/C) in GTEx Adipose Subcutaneous. The absolute effect size of the first and the second eQTLs are 1.93 and 0.98 as measured by $\log_2$ aFC. Each dot represents observed expression in one individual, scaled relative to the expression at "LL|LL" genotype. The blue and red bars show model fits from our two-eQTL model, and a loglinear regression model of the two eQTL genotypes, respectively. Haplotypes are separated by "|" sign (e.g. HL|HH corresponds to the cases that one haplotype carries high and low expressed alleles of eVariant1 and eVariant2, respectively, and the other haplotype carries the higher expressed allele of both eVariants.). E) Samples from genotype groups heterozygous for both eQTLs from panel D collapsed together. F) The predicted (x-axis) and observed (y-axis) haplotype effect on eGene expression for eGenes with at least three individuals available in each of the two genotype arrangement classes (n=539). Red and blue lines show the LOWESS and the linear regression fits, respectively.

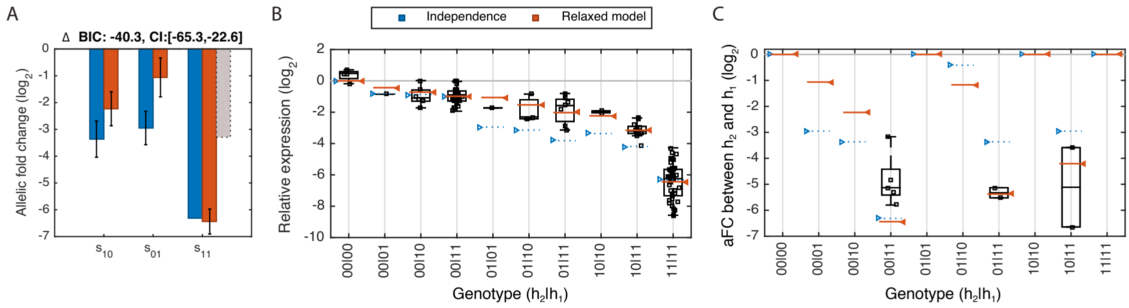

Supplemental Fig S7: An example of two eQTLs regulating the expression of the same gene that is not well described by their individual regulatory effects acting independently (eGene: *HLA-DQB1-AS1*; eVariant1: Chr6:32627082 A/G and eVariant2: Chr6:32609813 T/C; Tissue: LCL) A). Estimated log aFC associated with the alternative alleles for the first (s_{10}), and the second eSNP (s_{01}) individually, along with the estimated log aFC associated with co-occurrence of the alternative alleles (s_{11}). The independent regulation model is shown in blue, where s_{10} , s_{01} and s_{11} are estimated from the data with the constraint of $s_{11} = s_{10} + s_{01}$. The red bars show estimates from the alternative, relaxed model which allows for non-independence or epistatic-like interaction between the two eVariants, and s_{10} , s_{01} and s_{11} are estimated without assuming $s_{11} = s_{10} + s_{01}$. The support for non-independent effects comes from the difference between this estimated s_{11} to the sum of s_{10} and s_{01} from the relaxed model (gray dashed bar), which represents the expected joint effect of the two alternative alleles had they acted independently. B) Relative expression of the eGene and the model fits for the different genotype classes. Each dot is the expression observed in one individual, and expression levels are shown relative to the all-reference genotype. The blue and red bars show best fits achieved with and without the regulatory independence assumption, respectively. The model assuming regulatory independence between the two eVariants fails to adequately describe the observed data as measured by significance of BIC difference. C) Expression of the second haplotype relative to the first haplotype shown for different genotype groups. The dots indicate the observed values in ASE data and the blue and red bars show predicted values from the model fitted on eQTL data (as shown in panel B) using regulatory independence and the relaxed model, respectively. Genotypes in panel B and C are labeled following the notation in **Figure 6**, and classes identical with regard to the *cis*-regulatory model are collapsed together in each panel.

Supplemental Table Legend

Supplemental Table S1: eQTL effect size estimates for all GTEx eGenes associated with two distinct eQTLs.
