# Supplementary material for: Quantifying the regulatory effect size of cis-acting genetic variation using allelic fold change
Source: Genome Res. 2017 Nov;27(11):1872–84. doi: 10.1101/gr.216747.116 (PMC5668944; doi:10.1101/gr.216747.116)
Supplement: Supplemental Material [file supp_gr.216747.116_Supplemental_Software_S1.zip › Supplemental_Software_S1/aFC/README.pdf]

aFC

allelic **F**old **C**hange

Calculates allelic Fold Change (aFC) using standard input files for fastQTL.

Please see our preprint in bioRxiv for details and benchmarking of the method.

Method developed by Pejman Mohammadi, software by Stephane E. Castel both in the Lappalainen Lab at the New York Genome Center and Columbia University Department of Systems Biology.

Runs on Python 2.7.x and has the following dependencies: pandas, statsmodels, scikits-bootstrap, NumPy.

Usage

Requires tabix indexed gzip compressed VCF file containing genotypes and BED file containing phenotypes, identical to the inputs of fastQTL, and a list of QTL to calculate aFC for. If provided, covariates will be regressed out of the phenotype values. Outputs the aFC and corresponding 95% confidence interval for each input QTL.

Arguments

Required

- **--vcf** - Tabix indexed and gzipped VCF file containing sample genotypes. See fastQTL for format details.
- **--pheno** - Tabix indexed and gzipped BED file containing sample phenotypes. See fastQTL for format details.
- **--qtl** - File containing QTL to calculate allelic fold change for. Should contain tab separated columns 'pid' with phenotype (gene) IDs and 'sid' with SNP IDs. Optionally can include the columns 'sid_chr' and 'sid_pos', which will facilitate tabix retrieval of genotypes, greatly reducing runtime.
- **--geno** - Which field in VCF to use as the genotype. By default 'GT' = genotype. Setting to 'DS' will use dosage rounded to the nearest integer (IE 1.75 = 2 = 1|1).
- **--chr** - Limit to a specific chromosome.
- **--log_xform** - The data has been log transformed (1/0). If so, please set --log_base.
- **--o** - Output file.

Optional

- **--cov** *()* - Covariates file. See fastQTL for format details.

- **--matrix_o** *()* - Output the raw data matrix used to calculate aFC for each QTL into the specific folder.
- **--boot** *(100)* - Number of bootstraps to perform for effect size confidence interval. Can be set to 0 to skip confidence interval calculation, which will greatly reduce runtimes.
- **--ecap** *(log2(100))* - Absolute aFC cap in log2.
- **--log_base** *(2)* - Base of log applied to data. If other than 2, data will be converted to log2.

Output File

- 1 - **sid** - Variant ID.
- 2 - **pid** - Phenotype (gene) ID.
- 3 - **log2_aFC** - allelic Fold Change in log2.
- 4 - **log2_aFC_lower** - Lower estimate of 95% confidenace interval of log2(aFC).
- 5 - **log2_aFC_upper** - Upper estimate of 95% confidenace interval of log2(aFC).
